# Extended wakefulness alters the relationship between EEG oscillations and performance in a sustained attention task

**DOI:** 10.1111/jsr.14230

**Published:** 2024-05-05

**Authors:** Sophia Snipes, Elias Meier, Simon Accascina, Reto Huber

**Affiliations:** ^1^ Child Development Centre University Children's Hospital Zürich, University of Zürich Zurich Switzerland; ^2^ Neural Control of Movement Lab Department of Health Sciences and Technology Zurich Switzerland; ^3^ Independent Researcher; ^4^ Sleep & Health Zürich University of Zürich Zurich Switzerland; ^5^ Department of Child and Adolescent Psychiatry and Psychotherapy, Psychiatric Hospital University of Zürich Zurich Switzerland

**Keywords:** alpha, EEG, lapses, lateralised attention task, sleep deprivation, theta

## Abstract

During drowsiness, maintaining consistent attention becomes difficult, leading to behavioural lapses. Bursts of oscillations in the electroencephalogram (EEG) might predict such lapses, given that alpha bursts increase during inattention and theta bursts increase with time spent awake. Paradoxically, however, alpha bursts decrease with time awake and theta bursts increase during focussed attention and cognitive tasks. Therefore, we investigated to what extent theta and alpha bursts predicted performance in a sustained attention task, either when well rested (baseline, BL) or following 20 h of extended wakefulness (EW). High‐density EEG was measured in 18 young adults, and the timing of bursts was related to trial outcomes (fast, slow, and lapse trials). To increase the likelihood of lapses, the task was performed under soporific conditions. Against expectations, alpha bursts were more likely before fast trials and less likely before lapses at baseline, although the effect was substantially reduced during extended wakefulness. Theta bursts showed no significant relationship to behavioural outcome either at baseline or extended wakefulness. However, following exploratory analyses, we found that large‐amplitude theta and alpha bursts were more likely to be followed by lapse trials during extended wakefulness but not baseline. In summary, alpha bursts during baseline anticipated better trial outcomes, whereas large‐amplitude theta and alpha bursts during extended wakefulness anticipated worse outcomes. Therefore, neither theta nor alpha bursts maintain a consistent relationship with behaviour under different levels of overall vigilance.

## 1 INTRODUCTION

Sleepiness can be deadly. Being awake for 24 h has been found to deteriorate driving performance more than legal alcohol limits (Lowrie & Brownlow, 2020) and up to 20% of road traffic accidents have been attributed to insufficient sleep (Gibbings et al., 2022). While multiple cognitive systems are likely compromised during sleepiness, the most affected seems to be sustained attention (Lo et al., 2012). Laboratory tests of sustained attention such as the psychomotor vigilance task (PVT) reliably capture increases in behavioural lapses with time spent awake, circadian rhythm, and even cumulative sleep restriction (Basner & Dinges, 2011; Graw et al., 2004; Van Dongen et al., 2003). Given the role sleepiness can have on health and safety, there is a justifiable interest in identifying the neural mechanisms leading to these behavioural lapses.

One of the most notable features of brain activity are bursts of oscillations in the EEG, reflecting different states of vigilance. Theta oscillations (4–8 Hz) have been associated with sleepiness (Aeschbach et al., 1997; Finelli et al., 2000; Kaida et al., 2006), becoming larger and more frequent with time spent awake (Snipes et al., 2023). However, theta activity also increases during cognitive tasks and focussed attention (Brandmeyer & Delorme, 2018; Jensen & Tesche, 2002; Mitchell et al., 2008). This contradiction remains unresolved (Snipes et al., 2022). Similarly, alpha activity (8–14 Hz) is increased during inattention and relaxed wakefulness, anticipating behavioural lapses and tracking within‐session fluctuations in vigilance (Barry et al., 2007; Huang et al., 2007; Klimesch et al., 1998; Makeig & Jung, 1996). However, alpha activity decreases with time spent awake (Cajochen et al., 2002; Snipes et al., 2023). These discrepancies suggest that theta and alpha oscillations may relate to behaviour differently during elevated levels of sleepiness.

Such oscillations have typically been quantified using spectral power (Cajochen et al., 2002; Klimesch et al., 1998) or as single waves using amplitude thresholds (Andrillon et al., 2021; Bernardi et al., 2015; Fattinger et al., 2017). However, by using EEG burst detection with cycle‐by‐cycle analysis (Cole & Voytek, 2019), we found that the overall quantity of oscillations and their amplitudes can change independently (Snipes et al., 2023): alpha bursts become less frequent with time awake, whereas their amplitudes increase. Likewise, aperiodic background activity can change independently of oscillatory activity (Donoghue et al., 2020). Therefore, changes in spectral power are not sufficiently specific indicators of changes in the occurrences of oscillations.

**FIGURE 1:**
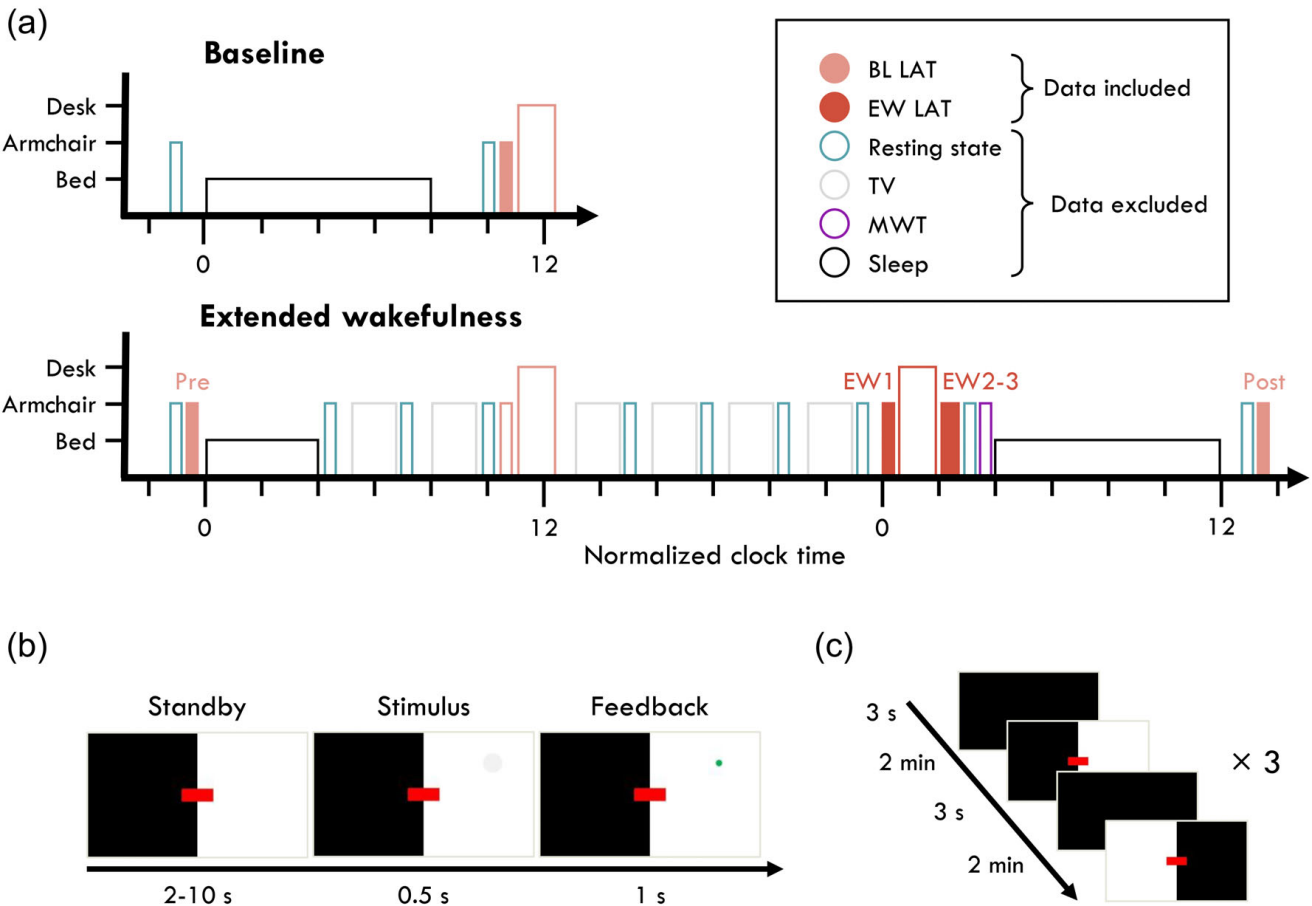
Study design. (a) Experiment schedule. Each block indicates an EEG recording session. Filled blocks indicate data analysed in this paper. Colour indicates the activity participants engaged in: grey, watching TV; turquoise, the resting state recordings (analysed in Snipes et al. (2023)); peach, baseline (BL) task blocks; red, extended wakefulness (EW) task blocks; purple, the maintenance of wakefulness test (MWT); black, sleep. The height of each block indicates the experimental condition in which data were collected: short, lying in bed; medium, seated in a comfortable armchair with foot and backrest; tall, seated at a desk, analysed in Snipes et al. (2022). The LAT and PVT were performed under soporific conditions in the armchair (data included), and additionally once in each task block at the desk (data not included). Brief empty spaces indicate transition periods allowing for delays. Six longer breaks were included prior to each TV block in which participants were provided with meals. Circadian time was normalised across participants to their habitual bedtime. Participants at baseline and during the recovery night were free to wake up when they wished, and at the beginning of the extended wakefulness period they were woken up after 4 h of sleep. Armchair tasks were performed in counterbalanced order with the desk tasks, and tasks within each block were randomised and counterbalanced for each participant. (b) A trial of the Lateralised Attention Task (LAT). Participants had to fixate on the red rectangle, and every 2–10 s, a grey circle would appear somewhere in the white area. If they pressed a button before the stimulus completely shrank away, it would flash green as positive feedback. (c) The 12 min LAT consisted of six blocks, alternating between the left and right screen being illuminated. Brief pauses separated the switch.

**FIGURE 2:**
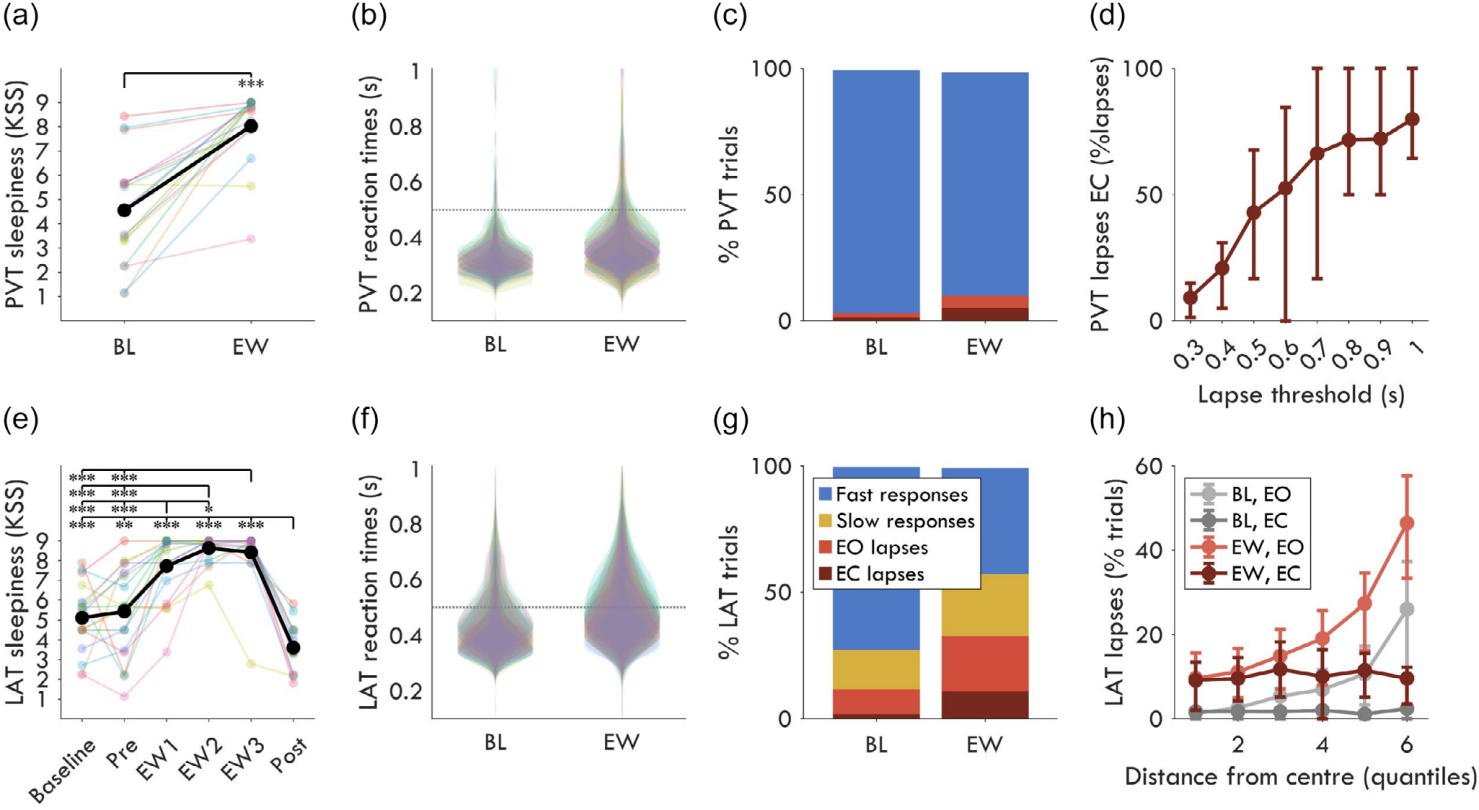
Behavioural outcome measures of the PVT (top row) vs the LAT (bottom row). (a) Subjective sleepiness recorded after the psychomotor vigilance test (PVT) using the Karolinska Sleepiness Scale (KSS). Baseline (BL) PVT was recorded after the baseline night of sleep, counterbalanced with the LAT. The extended wakefulness (EW) session was performed after ~20 h awake. KSS scores were recorded on a continuous scale with nine labels, from “1: extremely alert”, to “5: neither alert nor sleepy”, to “9: fighting sleep”. Thin coloured lines indicate values from individual participants, the thick black line is the group average. A paired *t*‐test (*α* = 5%) was conducted between BL and EW (*N* = 18, *t* = −6.37, *p* < 0.001, *g* = 1.79). (b) Reaction times during the PVT. Each coloured “violin” represents the distribution of an individual participant (*N* = 18). The dotted horizontal line at 0.5 s marks the threshold over which the trial was considered a lapse. (c) Average PVT trial outcomes (same legend as G) with lapses split by whether eyes were open (EO lapses, red) or closed (EC lapses, maroon). Figure only includes participants with data in both sessions (*N* = 13). EC fast trials are not included in the bar graph (they are the sliver of whitespace at the top). (d) Percentage of PVT lapses that are with EC, depending on the RT threshold used to define lapses (*N* = 9). Error bars indicate interquartile range around the average. The higher the RT threshold, the more lapses are due to EC. Anderson et al. (2010) performed a similar analysis, although they found 10% of lapses were with EC at 0.5 s cutoff, which only increased to 90% after ~2 s. This difference may be due to our soporific conditions. (e) Same as (a) for the LAT (*N* = 18). KSS scores for each LAT recording are provided separately, although all later analyses pool Baseline, Pre, and Post as BL sessions, and EW1, EW2, and EW3 as EW sessions. Baseline and EW1 LAT sessions were counterbalanced with the PVT. Paired *t*‐tests were conducted between all recordings, correcting with FDR (false discovery rate) for multiple comparisons. Stars indicating statistical significance such that: *p < 0.05, **p < 0.01, ***p < 0.001. (f) Same as B for LAT (*N* = 18), although the LAT was performed three times for each session block instead of just once. N.B: BL recordings are pooled from three different days, whereas EW recordings were within the same 3–4 h timespan, and therefore also include time‐on‐task effects (Doran et al., 2001). (g): Same as (b) for the LAT (*N* = 17). The LAT distinguishes *slow* trials as 0.5 s < RT <1 s (yellow) and *lapses* as trials where no response was given. (h) Percentage of LAT trials that are lapses, split into six quantiles based on the distance from the fixation point, such that 1 is closest and 6 is furthest (*N* = 17). 100% indicates all trials in that quantile were lapses (either EO or EC). Error bars indicate the interquartile range.

In this study, we investigated the relationship between EEG oscillations and behavioural outcomes to determine whether the presence of theta or alpha bursts predicted behavioural lapses in a visual attention task, and whether the relationship differed when well‐rested or following extended wakefulness (experiment design in Figure 1a). Participants performed the Lateralised Attention Task (LAT; Figure 1b,c), an adaption of the PVT which dissociates between slow responses and total response omissions, i.e. lapses (Figure 2). Based on previous findings, we expected alpha bursts to be more likely around lapse trials at least when well‐rested (Huang et al., 2007; Makeig & Jung, 1996), and theta bursts to be more likely around lapse trials at least during extended wakefulness (Bernardi et al., 2015; Vyazovskiy et al., 2011).

**FIGURE 3:**
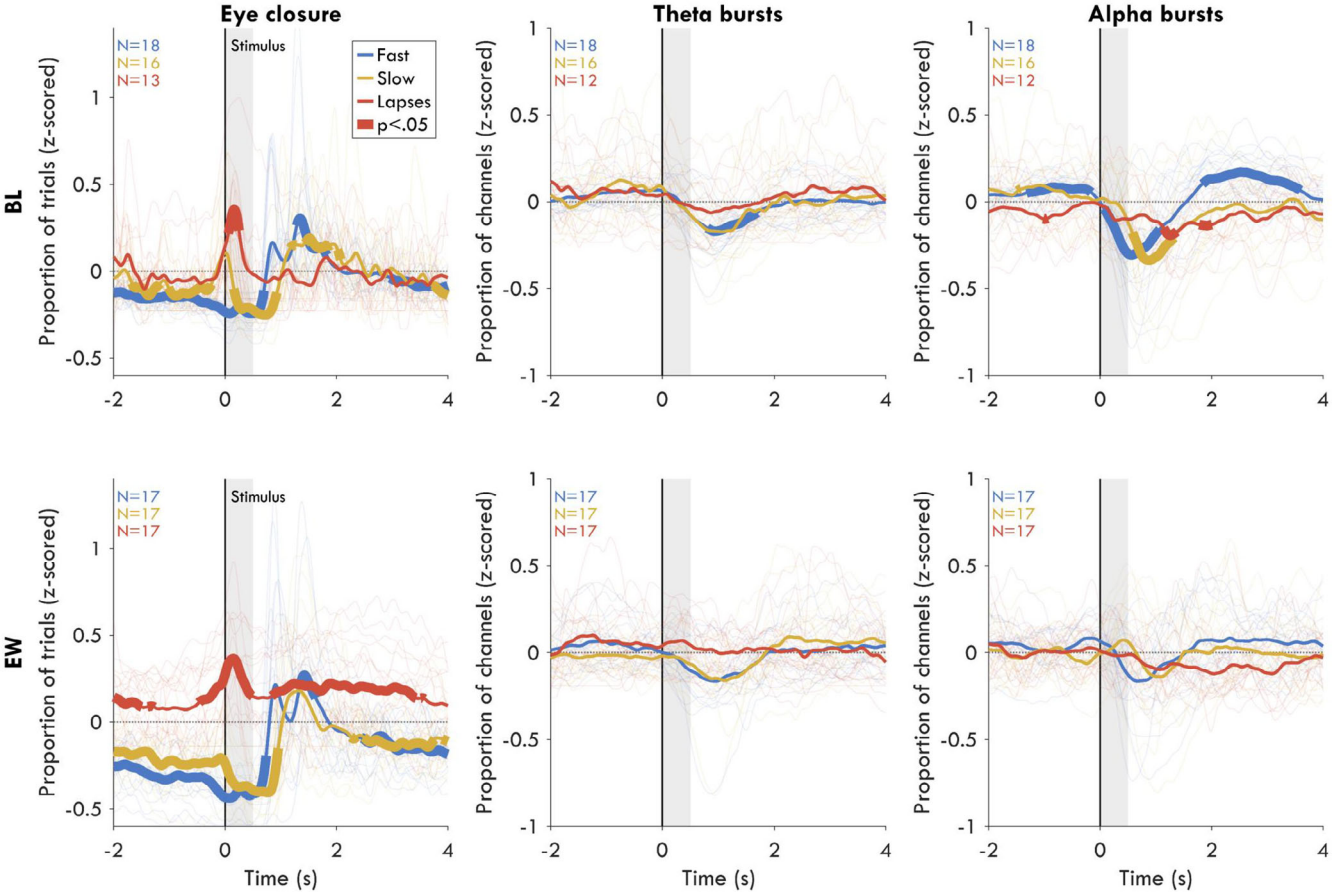
Distribution in time of bursts and eyes‐closed, locked to stimulus onset, split by trial outcome. Top row are BL recordings, bottom row are EW recordings. Time courses were smoothed using the lowess method over 0.2 s. Left column: The proportion of trials with eyes closed for each outcome type, normalised (*z*‐scored) to the session block average of eyes closed. Values at 0 (dotted horizontal line) indicate that eye‐closure was not more or less likely than average at that time point for that trial outcome. The thick vertical line represents stimulus onset, and the grey patch indicates the time in which the stimulus was visible. Light thin coloured lines represent individual averages, medium lines indicate the group average, and thick segments reflect time points in which the difference from average was statistically significant, with *p* < 0.05, FDR corrected for multiple comparisons. Sample sizes are indicated in the top left. Middle column: The proportion of channels with a theta burst (4–8 Hz), averaged across trials for each trial outcome, normalised to the average proportion of channels with a theta burst in that session. Trials and time points with eyes closed were excluded. Right column: Same for alpha bursts (8–14 Hz).

**FIGURE 4:**
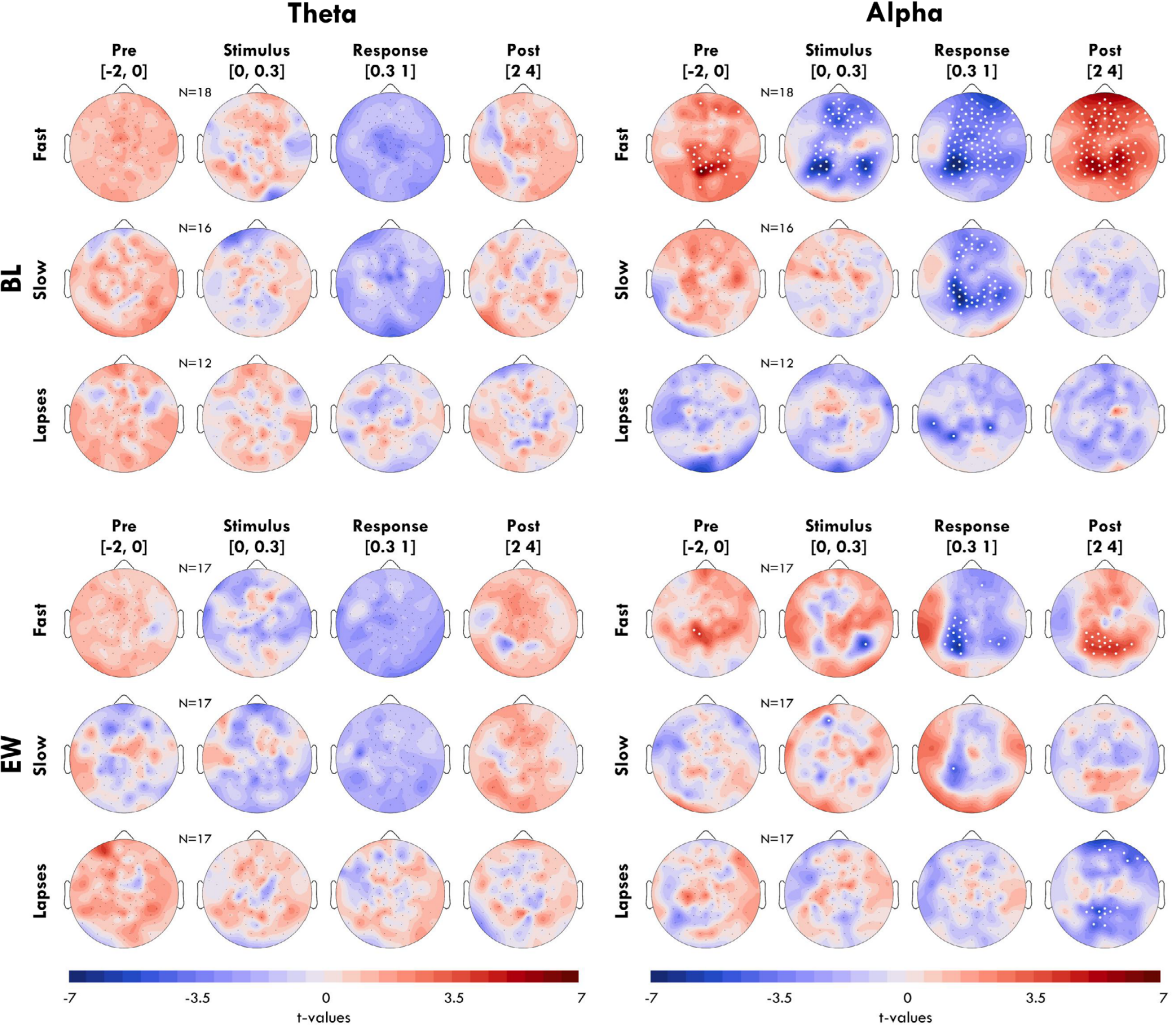
Topography of bursts per trial type and time window. Each topography (schematic view from above of the EEG net, nose pointed up) compares the likelihood of a burst at each channel (black dots) within a given window around the stimulus (columns; the window is in seconds from stimulus onset written in brackets), depending on whether the trial resulted in a fast response, slow response, or a lapse. The likelihood of a burst (i.e. the proportion of trials containing a burst) is compared with the overall likelihood of bursts in that frequency band in that channel in the entire session block of LAT recordings, quantified as *t*‐values and represented with colour, such that red indicates more bursts in that band in that channel in that time window compared with the session blockaverage. White dots indicate statistically significant differences, with *p* < 0.05. FDR correction was applied for each topography. Sample size is indicated for each trial outcome. Trials with eyes closed were excluded.

To test whether bursts predicted lapses, we investigated both the likelihood of bursts in time (Figure 3) and across channels (Figure 4) around stimulus onset according to trial outcome. We expected that bursts could be associated to lapses in two ways: they could be more likely exactly when the stimulus was present, indicating that bursts disrupted processing and responding to the stimulus; or they could be uniformly more likely in the seconds around lapse trials, indicating a general marker for a non‐vigilant state (Makeig & Jung, 1996) even if the bursts themselves do not directly cause the lapse. By analysing the changes in burst occurrences with high temporal resolution, we could determine the direction of causality. By analysing changes in topography, we had greater sensitivity to local effects.

## 2 METHODS

Different data from this experiment have previously been reported by Snipes et al. (2022) where the overall study design, participant selection, and EEG preprocessing was established, and by Snipes et al. (2023) where the burst detection method was developed and reported.

### 2.1 Participants

Eighteen participants completed the experiment. University student applicants were screened for good health, good sleep quality, and at least some sleep deprivation vulnerability. The mean age was 23 ± 1 years old, 9 were female, 3 were left‐handed, all had normal or corrected‐to‐normal vision, and self‐reported no hearing impairments. Further details can be found in Snipes et al. (2022), and the screening questionnaire is provided as Data S2. Data collection was conducted according to Swiss law and the principles of the Declaration of Helsinki, with Zurich cantonal ethics approval BASEC‐Nr. 2019–01193.

### 2.2 Experiment design

The full experiment schedule is depicted in Figure 1a. Participants came to the laboratory in two bouts: baseline, and extended wakefulness. During the baseline, participants went to bed at their habitual bedtime, and were free to wake up whenever they chose. On average they slept 8.0 ± 0.5 h. In the morning, they had breakfast and waited at least 40 min before beginning task recordings. During the extended wakefulness bout, participants slept only 4 h, were kept awake 24 h, alternating between watching TV, rest recordings, and breaks. We refer to this as a *4/24 extended wake* paradigm. All task recordings were performed under soporific conditions: seated in an armchair with footrest and headrest, lights turned off, and the task projected onto a wall. Subjective sleepiness was determined after each recording with the Karolinska Sleepiness Scale (KSS) (Åkerstedt & Gillberg, 1990).

#### 2.2.1 The lateralised attention task

The LAT is a 12 min visual–spatial reaction time task, modelled after the PVT. Six blocks (2 min each) alternated between having the left or right visual hemifield in white, and the other in black (Figure 1b,c). Participants had to maintain fixation on a red rectangle and covertly attend to the white half of the screen. Every 2–10 s a feint grey circle would appear in any location of the illuminated hemifield and shrink away completely within 0.5 s. Participants needed to press a button before the circle disappeared, in which case the circle would freeze and flash green. Responses earlier than 0.1 s were considered false alarms. Reaction times (RTs) from 0.1 to 0.5 s were considered *fast*, and from 0.5 to 1 s were considered *slow*. Trials were considered *lapses* otherwise. After five consecutive lapses, an alarm would sound to wake up the participant. During the delay periods, 50 ms pink noise tones were presented every 1.5–5 s at ~50 dB. Participants were instructed to ignore these tones. These were not related to the main research question of this manuscript. The task was implemented with PsychoPy (v3.2.4) and is available at https://github.com/snipeso/LAT.

The LAT was performed three times under well‐rested baseline conditions (BL: morning after baseline sleep, evening before extended wakefulness, and morning after recovery sleep), and three times after extended wakefulness (EW: once in the counterbalanced block, twice more afterwards). Most participants had over 300 trials per session block. One participant completed only 1/3 of the EW LAT recordings, another participant completed only 2/3 EW LAT recordings. All others had 3 BL and 3 EW EEG recordings. For each analysis for each participant there had to be at least 15 trials per trial outcome.

#### 2.2.2 The psychomotor vigilance task (PVT)

A 10 min PVT was performed the morning after baseline sleep, and once after extended wakefulness. This involved a counter appearing in the centre of the screen every 2–10 s, and participants were instructed to push a button as fast as possible to stop the counter. They were given their RTs as feedback. PVT lapses were trials for which RTs were >0.5 s (Basner & Dinges, 2011). If participants did not respond within 5 s, an alarm sounded to wake them up.

### 2.3 EEG analysis

EEG data were recorded at 1000 Hz with Cz reference, using BrainAmp amplifiers and 128‐channel EGI Geodesic nets. Preprocessing and data analysis were done using EEGLAB (Delorme & Makeig, 2004) and custom MATLAB scripts (R2019b, R2022b, R2023a, R2023b). Data were low‐pass filtered at 40 Hz, downsampled to 250 Hz, and high‐pass filtered at 0.5 Hz. Major artefacts were identified visually, and physiological artefacts (eye movements, heartbeat, muscle activity) were removed with independent component analysis (ICA). The final channel count was 123. Further details are provided in Snipes et al. (2022).

Bursts were detected using cycle‐by‐cycle analysis implemented in MATLAB with adaptations published previously (Snipes et al., 2023). To identify bursts, first clean EEG data were filtered in narrow overlapping bands 4 Hz wide, from 2 to 16 Hz, and for each band, zero‐crossings were identified. Then in the broadband filtered data, positive and negative peaks were found between zero‐crossings as maximum and minimum voltages; a cycle was then defined from positive‐to‐positive peak. Each cycle was characterised by properties of the broadband filtered signal, and a minimum number of consecutive cycles had to have properties meeting a set of criteria to be classified as a burst (e.g. monotonicity, period consistency). Bursts were then sorted as theta and alpha based on the mean negative peak‐to‐peak period. A detailed explanation of the algorithm, specific burst criteria, and validation of the burst detection are provided in Data S1. The analysis code is available on https://github.com/snipeso/Lapse-Causes, and the burst detection as a toolbox https://github.com/HuberSleepLab/Matcycle.

#### 2.3.1 Burst likelihood by trial outcome

To quantify whether a burst was more likely to occur at a given time point for a given trial outcome (Figure 3), for each trial the proportion of channels with a burst at every time point was calculated, then averaged across trials, separately for theta and alpha. N.B. this does not discriminate between the globality of any given burst, and the total number of distinct bursts co‐occurring. “Burst likelihood” is used for simplicity and consistency, however, results from Figure 3 not present in Figure 4 could also be due to changes in burst globality. Data were epoched around the stimulus trigger sent from the task computer to the EEG. Time points containing artefacts were removed. Trials were excluded if more than 50% of the data was missing. If there were fewer than 15 trials with clean data at any time point, those time points were interpolated. If there were such gaps larger than 20% of the trial window, then this trial outcome was excluded for that participant. The proportion of channels with bursts for each trial outcome was then *z*‐scored to the mean and standard deviation of the proportion of channels with bursts recorded across the entire EEG session block. The *z*‐scoring was done to have normally distributed values and to reduce the influence of participants with substantially more oscillatory activity.

To determine the topography of the likelihood of bursts across outcomes (Figure 4), the proportion of trials containing a burst was calculated for each channel at every time point, and this was then averaged within the four windows (Pre: −2‐0 s; Stimulus: 0–0.3 s; Response 0.3–1 s; Post: 2–4 s). These averages were then compared with the average proportion of time points with bursts for a given channel in the entire session block.

#### 2.3.2 Lapse likelihood by burst amplitude

To explore the relationship between burst amplitudes and behavioural outcome, all bursts from −1 to 0 s before stimulus onset were identified, and the average amplitude of all cycles was calculated within this window for each burst. Bursts were then sorted into 10 amplitude quantiles within each session block. The proportion of trials resulting in a lapse were then calculated for each quantile. These values across quantiles were then *z*‐scored for each participant. We excluded trials for which eyes were closed during the stimulus window.

#### 2.3.3 Time‐frequency analysis

**FIGURE 5:**
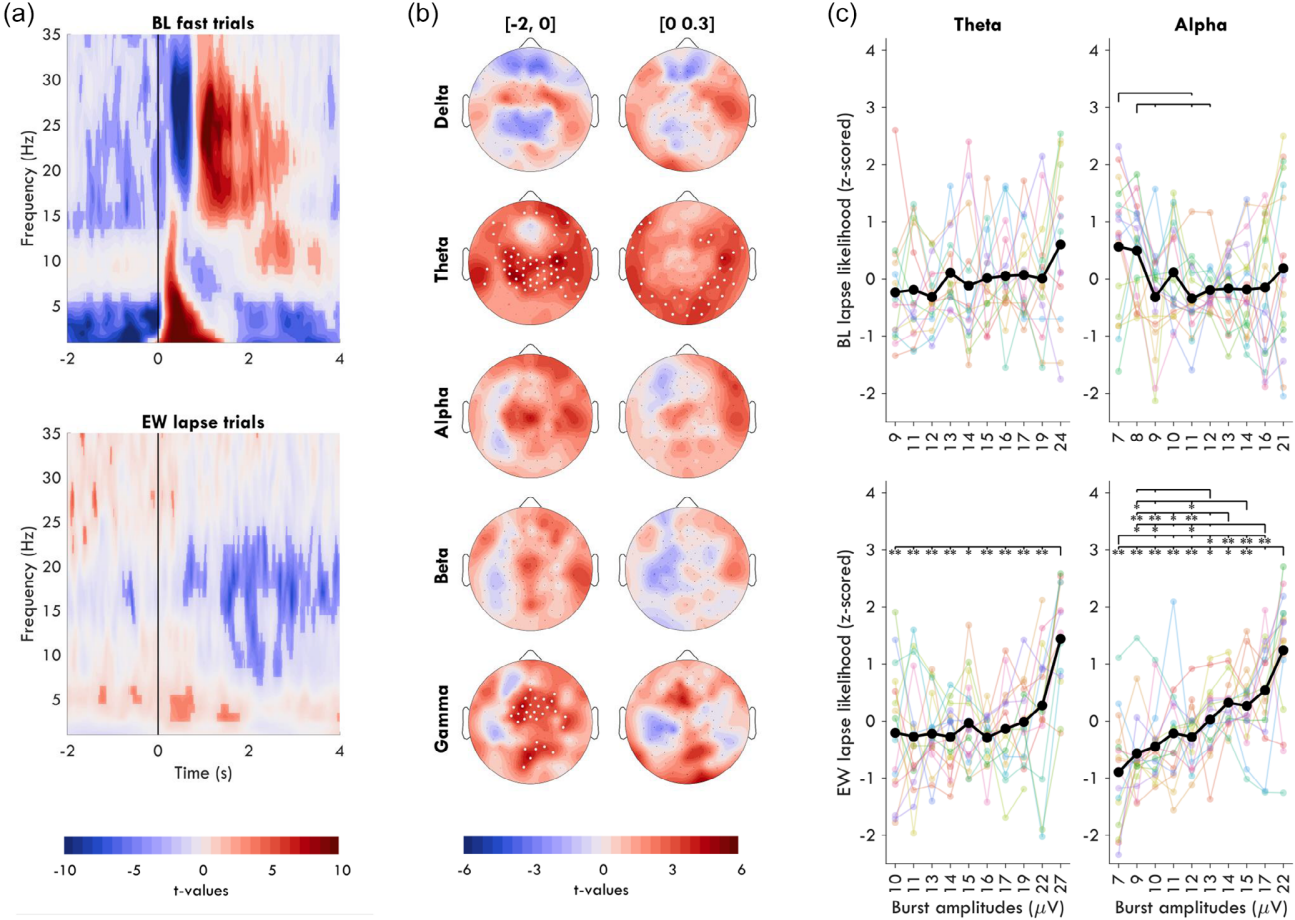
Exploratory analyses. (a) Time‐frequency power analysis locked to stimulus onset for BL fast trials (top) and EW lapse trials (bottom). All other trials from BL and EW are in Suppl. Figure 3. Power values were log‐transformed and compared with the session average, then averaged across all channels, with colour reflecting *t*‐values such that red indicates higher than average power for that frequency at that time point. A pale mask covers values that were not statistically significant following FDR correction. Trials were not corrected for eye‐closures. (b) Average power from the time‐frequency analysis, from the Pre window (left) and Stimulus window (right), split by frequency bands delta (1–4 Hz), theta (4‐8 Hz), alpha (8–14 Hz), beta (15–25 Hz), and gamma (25–30 Hz). Trials were corrected for eye‐closures. FDR correction was applied to each topography. (c) The likelihood of a lapse following bursts of different amplitudes. All bursts that occurred 1 s before a stimulus were binned into 10 quantiles by amplitude and the proportion of trials resulting in a lapse was determined for each quantile. X tick labels indicate the average amplitude for each quantile; note that each quantile increases in amplitude from BL to EW, but the distributions largely overlap. Values were then *z*‐scored within each session block for each participant. Paired *t*‐tests were conducted between all quantiles, FDR corrected, with stars indicating *p*‐values such that: *p < 0.05, **p < 0.01, ***p < 0.001. For theta Q1 to Q10 during BL: *N* = 15, *t* = −1.68, *p*_fdr_ = 0.832, *g* = 0.66. Theta Q1 to Q10 EW: *N* = 14, *t* = −4.34, *p*_fdr_ = 0.006, *g* = 1.63. Alpha Q1 to Q10 BL: *N* = 18, *t* = 0.68, *p*_fdr_ = 0.758, *g* = −0.28. Alpha Q1 to Q10 EW: *N* = 17, *t* = −5.72, *p*_fdr_ = 0.001, *g* = 2.03.

An exploratory time‐frequency analysis was conducted using Morlet wavelets for frequencies between 1 and 35 Hz, using 3 to 15 cycles logarithmically spaced. Power was log transformed. Data were then epoched around stimulus onset with the same procedure as for bursts, excluding artefacts. Time points with eye‐closure were initially included (Figure 5a), then the analysis was repeated excluding eye‐closures (Figure 5b). Average power across the entire session block was subtracted from each time point of each trial. Trials were not otherwise baseline corrected. Trials were averaged by trial outcome, and one‐sample *t*‐tests were used, thus identifying deviations from average spectral power for each trial outcome.

### 2.4 Eye‐tracking and controlling for eye‐closure

To exclude lapses which were due to eye closure we measured ocular activity with Pupil Core “glasses” from Pupil Labs. Pupil Player software estimates pupil diameter and provides a confidence value; we considered confidence <0.5 to be eyes closed. Multiple technical failures and poor data quality resulted in data loss such that six participants had only two BL eye‐tracking recordings, one participant had only one EW eye tracking, and one participant had no EW eye‐tracking. Periods with eye‐closure were treated the same as if they were artefacts in the EEG and thus excluded. Additionally, trials were removed if the eyes were closed for more than 50% of the stimulus window (because the lapse would simply be due to eye‐closure).

### 2.5 Statistics

All statistical comparisons were paired/one‐sample *t*‐tests, with false discovery rate (FDR) correction applied within each figure using the Benjamini‐Hochberg method (Benjamini & Hochberg, 1995). Effect sizes were calculated as Hedge's *g*. Post‐hoc statistical power analyses (MATLAB function *sampsizepwr*) with *α* = 0.05 and 1−*β* = 0.8 indicated that for 18 participants there was power for *g* >0.68, and with 10 participants there was power for *g* >0.94. When reporting mean values in the text, instead of including standard deviations, the interquartile range is indicated (25% and 75% of participants’ values).

## 3 RESULTS

### 3.1 The experimental paradigm successfully increased sleepiness and behavioural lapses

First, we validated the experimental paradigm and compared the novel LAT with the established PVT. Subjective sleepiness increased significantly with extended wakefulness during both the PVT (Figure 2a) and the LAT (Figure 2e). Reaction times were slower for the LAT (Figure 2f) compared with the PVT (Figure 2b), resulting in a larger proportion of trials with RTs >0.5 s. The average percentage of LAT lapse trials was 12% [interquartile range: 5, 19] at BL, and 33% [22, 44] during EW. By comparison, PVT lapses were 3% [1, 4] of BL trials, and 10% [2, 14] of EW trials (Figure 2c). Only 4% [1, 6] of PVT EW trials were eyes‐open lapses, whereas 22% [13, 30] of LAT trials were eyes‐open lapses. The proportion of eyes‐closed PVT lapses decreased with decreasing RT thresholds (Figure 2d), however, these increasingly encompass “sluggish” responses rather than attention lapses per se. Instead, the distributions of LAT RTs were well within the 1 s cutoff for slow trials, with 99% of RTs <0.83 s. Therefore, the LAT captured substantially more eyes‐open behavioural lapses than the PVT and can separate slow responses from lapses. This makes the LAT an appropriate task for associating neural signatures to attention lapses.

Because stimuli appeared at any distance from the fixation point during the LAT, it could be that most lapses occurred at the edge of participants’ visual field. To check if this was the case, trials were divided into six quantiles based on radial distance from the fixation point (Figure 2h). At BL, considering only EO trials, the closest quantile had 1.3% [0.0, 1.8] of trials as lapses and the furthest had 26.0% [10.4, 37.3]. Therefore, while distance was clearly a major contributor of lapses at BL, there was no distance after which stimuli were completely missed and were thus outside of the field of view. In other words, distance from fixation increased the chances of a lapse but did not determine one.

### 3.2 Alpha bursts reflect higher vigilance at baseline

To evaluate whether bursts could be either causally related to lapses or a general marker of reduced vigilance, we investigated the relationship in time between bursts and trial outcome. As a proof of principle for this approach, we first performed this analysis for eye‐closures, an obvious driver of behavioural lapses (Figure 3, left). At BL, eye‐closures were significantly more likely only during the stimulus window (max time: 0.16 s, *N* = 13, *t* = 4.53, *p*_fdr_ = 0.002, *g* = 1.70), reflecting coincidental blinks. During EW, the main difference from BL was that eye‐closures were more likely in the seconds prior to lapses (max time: −1.89 s; *N* = 17, *t* = 3.20, *p*_fdr_ = 0.011, *g* = 1.06) and after lapses (max time: 1.90 s; *N* = 17, *t* = 3.68, *p*_fdr_ = 0.005, *g* = 1.22), reflecting prolonged periods with eyes closed, very likely microsleeps (Hertig‐Godeschalk et al., 2020; Ong et al., 2013). Thus, eye‐closure both directly causes lapses and acts as a general marker of reduced vigilance.

For theta bursts at BL during lapse trials (Figure 3, middle), there was no time point for which bursts were more likely than average either before (max *t*: −2.00 s; *N* = 12, *t* = 1.95, *p*_fdr_ = 0.321, *g* = 0.76), during (max *t*: 0.06 s; *N* = 12, *t* = 0.75, *p*_fdr_ = 0.790, *g* = 0.29), or after the stimulus appeared (max *t*: 3.60 s; *N* = 12, *t* = 1.85, *p*_fdr_ = 0.366, *g* = 0.72). The only significant effects for theta at BL were decreases after the stimulus window of fast trials (max *t*: 1.45 s; *N* = 18, *t* = −9.31, *p*_fdr_ <0.001, *g* = −3.01). For theta bursts during EW, again there were no significant time points before (max *t*: −1.52 s; *N* = 17, *t* = 2.32, *p*_fdr_ = 0.305, *g* = 0.77), during (max *t*: 0.30 s; *N* = 17, *t* = 1.84, *p*_fdr_ = 0.453, *g* = 0.61), or after lapse trials (max *t*: 0.34 s; *N* = 17, *t* = 1.96, *p*_fdr_ = 0.393, *g* = 0.65). While on average there were still decreases in theta bursts after the stimulus, this effect was no longer significant during EW (max *t*: 1.39 s; *N* = 17, *t* = −3.24, *p*_fdr_ = 0.151, *g* = −1.08). Therefore, theta bursts did not significantly affect trial outcome and did not predict fluctuations in vigilance either when well rested or following extended wakefulness.

For alpha at BL (Figure 3, right) there were significantly *fewer* bursts before lapse trials (max *t*: −0.99 s; *N* = 12, *t* = −3.18, *p*_fdr_ = 0.034, *g* = −1.24) and fewer bursts after the stimulus window (max *t*: 1.90 s; *N* = 12, *t* = −3.42, *p*_fdr_ = 0.024, *g* = −1.33). Fewer alpha bursts were also present during the stimulus window, but the effect was not significant (max *t*: 0.27 s; *N* = 12, *t* = −1.90, *p*_fdr_ = 0.172, *g* = −0.74). Vice versa, alpha bursts were significantly more likely before fast trials (max *t*: −0.87 s; *N* = 18, *t* = 4.78, *p*_fdr_ = 0.003, *g* = 1.55). Across the stimulus window, the proportion of alpha bursts markedly decreased (max t: 0.35 s; N = 18, *t* = −5.15, *p*_fdr_ = 0.002, *g* = −1.67), corresponding to the well‐known event‐related desynchronisation of alpha (Pfurtscheller & Lopes da Silva, 1999). There was an additional positive rebound in alpha bursts after the trial. During EW, as for theta bursts, no time point showed significant differences in alpha bursts (*p* > 0.118). Therefore, alpha bursts reflected higher vigilance, but only when well‐rested.

In Figure 4 we plot the topographies of the effects from Figure 3, averaging across four time windows around the stimulus. No channel had significant differences in proportion of theta bursts either at BL or EW for any trial outcome. Alpha bursts at BL were significantly more likely before the stimulus in an occipital‐midline cluster (channel with maximum *t*‐value: 71). The subsequent decrease in alpha bursts was widespread (stimulus window: 42% of channels were significant; response window: 76%), peaking in frontal and lateral‐occipital channels. The rebound was likewise widespread (79%). Only a few isolated channels were significantly lower for alpha in the response window of lapse trials (max ch: 59). During EW, the effects observed for alpha bursts during fast trials were much more localised (Pre: 2%; Stimulus: 1%; Response: 16%; Post 15%). Instead, the decrease in alpha following lapse trials was more pronounced, but only in the 2–4 s after the stimulus (max ch: 67).

### 3.3 Large amplitude theta bursts anticipate more lapses during extended wakefulness

Given that the results in Figure 3 are opposite from those in previous publications for alpha activity (Huang et al., 2007; Makeig & Jung, 1996), we performed time‐frequency analyses to determine whether the effect was driven exclusively by the analysis method (burst detection) or the experimental paradigm (soporific conditions & LAT). We found that prior to fast trials at BL, spectral power was significantly lower in all frequencies *except* the alpha band, with the effect largest in the delta band (Figure 5a, top).

The time‐frequency analysis also revealed statistically significant differences in power around lapses during EW (Figure 5a, bottom) but not BL (Figure S3), contrasting our non‐significant findings based on burst detection. Therefore, we conducted exploratory analyses to find an explanation for this discrepancy. First, we looked at the topography of spectral power around EW lapses, excluding trials with eyes‐closed (Figure 5b). In the 2 s window before the stimulus (left column), central theta and gamma power was increased relative to the EW average. In the window during the stimulus (0–0.3 s; right column), only occipital and temporal channels had significantly elevated theta power.

A possible explanation for the discrepancy between the spectral power analysis and burst detection is that the *amplitudes* of oscillations contribute to the significant effect found in power. Therefore, we compared the likelihood of lapses for bursts of different amplitudes in the 2 s prior to stimuli (before stimulus‐induces changes to oscillations could occur). We found that during EW, the largest 10% of theta bursts had significantly greater chances of being followed by a lapse compared with all other quantiles (Figure 5c), increasing from 23% [15, 28] of trials as lapses for the smallest quantile to 33% [27, 40] for the largest. Despite no significant relationship between alpha power and EW lapses, alpha bursts showed a linear relationship between increasing amplitude and increasing likelihood of a lapse, passing from 23% [12, 34] to 29% [19, 37]. At BL, no significant difference across quantiles was found for theta. BL alpha had a trending higher lapse likelihood for the smallest bursts. Therefore, only during EW was there a relationship between the largest amplitude bursts and an increase in the likelihood of a behavioural lapse.

## 4 DISCUSSION

With this study, we evaluated whether there was a meaningful relationship between oscillation bursts and attention lapses, and whether the relationship differed when well rested or following extended wakefulness. By analysing the likelihood of bursts in time by trial outcome (Figure 3) we found that neither theta nor alpha bursts were more likely to occur during the stimulus window of lapse trials, either during BL or EW. We further found that alpha bursts were more likely before fast trials in central‐occipital channels, but only during BL (Figure 4). Through exploratory spectral power analyses, we found that theta power was elevated around lapses during EW (Figure 5a,b), contradicting the results observed when analysing bursts. We found that this could be explained by large‐amplitude theta bursts during EW, which were more likely to anticipate lapse trials than smaller bursts (Figure 5c).

An important observation derived from these results is that the majority of bursts that occur in the EEG do not directly cause lapses. If this had been the case, then the time courses for theta and alpha should have resembled those for eye‐closures (Figure 3, left). A plausible explanation for why most bursts do not impact behaviour is that oscillations are generated from task‐unrelated areas. Both theta and alpha oscillations have been associated with reduced fMRI BOLD activity (functional magnetic resonance imaging, blood oxygen level dependent), from the regions generating the oscillations (Scheeringa et al., 2008, 2012), and these sources were not involved in the ongoing task (Kirschfeld, 2005; Laufs et al., 2003; Michels et al., 2010; Rihs et al., 2007; Scheeringa et al., 2009; Snipes et al., 2022).

The exception may be large‐amplitude theta bursts during extended wakefulness. Theta power in occipital and temporal channels was significantly higher than average during the stimulus window of lapse trials (Figure 5b, right). The reason this may not have appeared in the burst analysis of Figure 3 is that the effect was driven by a minority of high‐amplitude bursts. We could not directly compare the effect of amplitude on trial outcome for bursts occurring during the stimulus window given the event‐related desynchronisation for fast trials, but we could do so for bursts in the 2 s before stimulus onset. Like this, we determined that the probability of a lapse was significantly higher following the largest 10% of theta bursts compared with all other amplitudes. Therefore, few large‐amplitude bursts can explain why average theta power was significantly elevated before lapse trials (Figure 5b, left) but not the proportion of bursts before lapses (Figure 4). These large amplitude theta bursts may be the same “local sleep” events driving lapses in sleep deprived rats (Vyazovskiy et al., 2011) and humans (Andrillon et al., 2021; Bernardi et al., 2015; Fattinger et al., 2017).

Unlike theta, alpha bursts were related to performance during BL but in the opposite direction from what we expected, anticipating faster trials. This directly contradicts previously published results identifying alpha power as a within‐session marker of reduced vigilance (Hanslmayr et al., 2011; Huang et al., 2007; Makeig & Jung, 1996; Sauseng et al., 2005). An explanation for these opposing results is that alpha and vigilance could follow a non‐linear relationship, such that low alpha characterises both extremely high and extremely low vigilance. Recent work by Pfeffer et al. (2022) found in fact that alpha activity and pupil diameter follow this inverted U pattern, such that both small pupils (indicating low vigilance) and large pupils (indicating high vigilance) are associated with lower alpha compared with intermediate values. It could be that previous studies were conducted under higher overall vigilance, from which drops in alertness (and performance) corresponded to increased alpha. Instead in our study, participants performed the task under deliberately soporific conditions of the task (dark room, armchair). Their average subjective sleepiness scores were in the middle of the KSS scale, indicating that they were “neither alert nor sleepy”. This would explain why for this study, further drops in alertness even at baseline resulted in *decreased* alpha. This inverted U relationship between alpha and vigilance could likewise explain why Kaida et al. (2006) found a positive correlation between alpha and subjective sleepiness when well rested, but Strijkstra et al. (2003) found a negative correlation across sleep deprivation. The fact that alpha decreases at all during sleep deprivation is also a clear indication that alpha cannot be a monotonic marker of vigilance.

In addition to alpha bursts no longer anticipating fast trials, during EW, alpha amplitudes showed a linear relationship to the increasing likelihood of a lapse (Figure 5c). This effect was absent during BL, despite overlapping distributions of alpha amplitudes. This suggests that whatever aspect of alpha activity could have been beneficial for performance at BL, it becomes detrimental during EW in a “dose‐dependent” manner. This further underscores the different relationship between bursts and behaviour during EW compared with BL.

### 4.1 Limitations

An important limitation of this study is sample size. With 18 participants, we only have enough power for medium‐large effect sizes. Therefore, non‐significant effects such as the proportion of theta bursts around lapse trials may simply be underpowered. Another important limitation is the burst detection itself. We found that the detected bursts accounted for the majority of theta periodic power and the totality of alpha power (Figure S2), indicating most oscillatory bursts were successfully detected, but we cannot estimate the proportion of false positives. These may have masked any significant relationships between true bursts and behaviour. Finally, our results could also be explained by circadian rhythms, which the extended wakefulness paradigm cannot dissociate from effects due to the buildup in sleep need with time spent awake.

## 5 CONCLUSION

Our results indicate that most EEG bursts do not contribute to behavioural lapses, supporting previous findings that localise both theta and alpha activity to task‐unrelated areas. The exception may be large‐amplitude theta bursts appearing during extended wakefulness. We further found a linear relationship between alpha amplitudes and lapse likelihood present during extended wakefulness but not baseline. This suggests that the increase in eyes‐open lapses during extended wakefulness may be driven not only by intrusions of lapse‐causing events, but a greater vulnerability to routine yet conflicting neuronal processes. Finally, while alpha has traditionally been considered a marker of inattention, our results show that sometimes the complete opposite can be true, with periods of high alpha anticipating better performance. This makes alpha activity an unreliable marker of vigilance, and in general our results demonstrate that the relationship between bursts and behaviour differs depending on underlying levels of vigilance.

## AUTHOR CONTRIBUTIONS

**Sophia Snipes:** Conceptualization; methodology; software; data curation; investigation; formal analysis; visualization; writing – original draft; writing – review and editing. **Elias Meier:** Investigation; writing – review and editing; data curation. **Simone Accascina:** Software; writing – review and editing. **Reto Huber:** Conceptualization; methodology; supervision; funding acquisition; resources; writing – review and editing.

## CONFLICT OF INTEREST STATEMENT

None of the authors have any competing interests.

## Supporting information


**DATA S1** Supporting Information.

**DATA S2** Supporting Information.

## Data Availability

The data that support the findings of this study are available from the corresponding author upon reasonable request.
