# Supplementary material for: Extended wakefulness alters the relationship between EEG oscillations and performance in a sustained attention task
Source: J Sleep Res. 2024 May 5;33(6):e14230. doi: 10.1111/jsr.14230 (PMC11596987; doi:10.1111/jsr.14230)
Supplement: Supplementary file 1 — DATA S1 Supporting Information. [file JSR-33-e14230-s001.pdf]

SUPPLEMENTARY INFORMATION

Burst detection criteria

Burst detection was performed using adaptations of cycle-by-cycle analysis (Cole & Voytek, 2019). Given that this is still a new approach under development, we opted to apply the same principles for tuning the burst detection algorithm as applied for machine learning: an independent training dataset was used to adapt parameters until bursts were detected that sufficiently matched visual inspection, and then those criteria were used without further changes to the testing dataset. The training dataset consisted of EEG recordings during baseline and extended wake in the same participants performing the PVT task, as well as a game and resting data. Given that the training data originated from the same participants as the testing data, these burst detection criteria may be “overfitted” to this set of participants. However, we recently applied the same criteria to a dataset of >160 participants 3 to 25 years old with comparable success (Snipes et al., 2024). The choice of multiple criteria sets was done to account for different possible bursting patterns observed in the data.

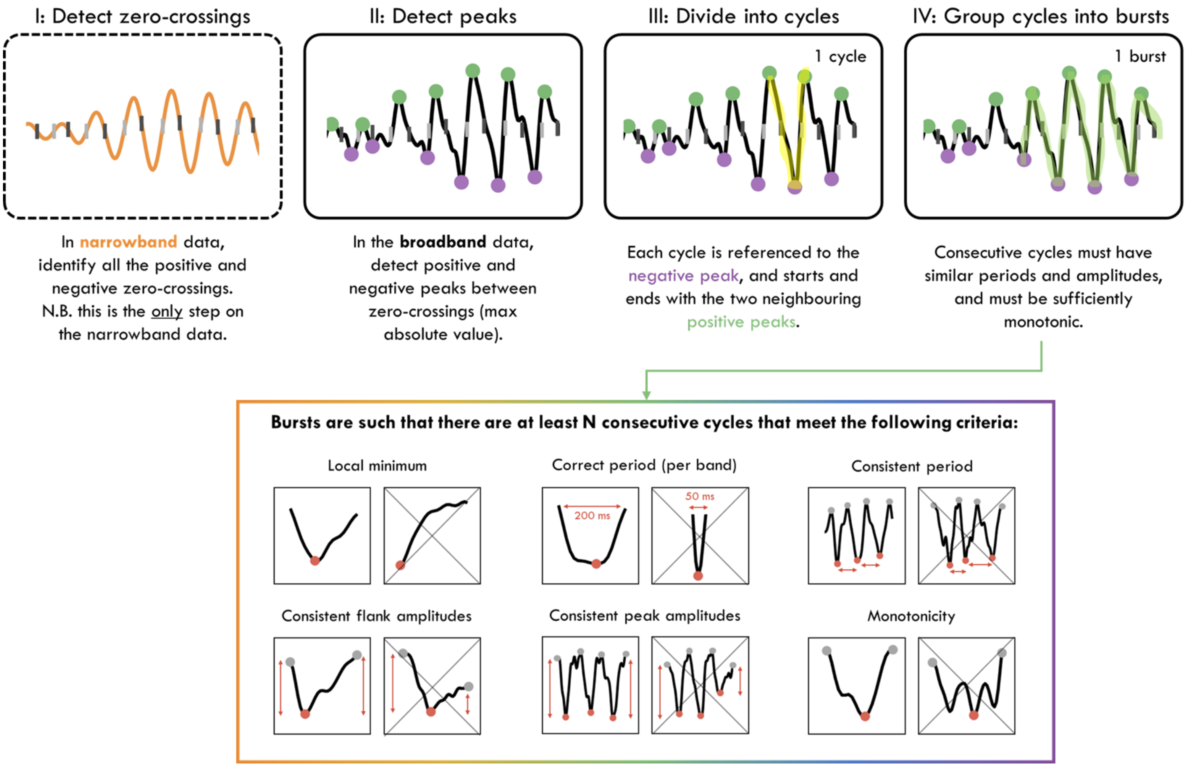

Figure 1: schematic of burst detection. The full figure is published in (Snipes et al., 2023). Not all criteria were used for each criteria set (see below).

Multiple criteria sets were used, on both the signal and its inverse, and so from bursts overlapping in time in the same channel, only the longest was kept. Bursts that were only present in one channel were also excluded.

Criteria set 1: at least 4 consecutive cycles had to have a period in the narrowband filtered range, similar consecutive periods (period consistency = .5), similar consecutive amplitudes (amplitude consistency = .4), similar rising and descending amplitudes (flank consistency = .5), they had to correlate with

neighboring cycles (shape consistency = .2),[1] a minimum proportion of *timepoints* that changed amplitude in the correct direction of the cycle (monotonicity in time = .4), a minimum proportion of the *amplitude* that changed in the correct direction of the cycle (monotonicity in amplitude = .4) and a maximum ratio of the largest segment in the incorrect direction (reversal ratio = .6; ratio of the largest reversal to the smallest cycle flank).

Criteria set 2: at least 3 cycles; period in the narrowband filter range; period consistency = .7; flank consistency = .3; amplitude monotonicity = .9. This captured bursts that were generally high-amplitude, clearly oscillatory, but possibly very brief.

Criteria set 3: at least 5 cycles; period consistency = .6; amplitude consistency = .6; amplitude monotonicity = .6; flank consistency = .6. This captured bursts that were more difficult to discriminate from the background activity if not for their long duration.

Bursts capture the majority of periodic power

The success of the burst detection was evaluated by comparing the power spectra of EEG data with bursts (intact) to the same EEG data once timepoints containing bursts had been removed (burstless). EEG spectral power captures both periodic (oscillations) and aperiodic activity (1/f "colored background noise") (Donoghue et al., 2020), so we first subtracted the aperiodic activity, what is known as "whitening" the power spectrum. Spectral power was calculated using Welch's method, with 8 s windows and 50% overlap. Participants were excluded if there was less than 1 minute of data without bursts. The power spectra were then smoothed with a lowess filter (2 Hz). Using the FOOOF toolbox (Donoghue et al., 2020) the aperiodic components were determined with data from 2 to 40 Hz, and then subtracted from each spectrum, the result of which is plotted in Figure 2B,D. Finally, power in the theta and alpha band were integrated for both the intact spectra and the burstless spectra. These values were quantified as the percent decrease in periodic power from intact to burstless data. Like this, a 100% decrease indicates that the entire periodic power in that frequency band was driven by the detected bursts, and therefore essentially all of the oscillatory activity was captured by the burst detection algorithm. This evaluation was done for the channels and recording sessions with the largest theta and alpha oscillatory activity in order to see the largest effect and to maximize the separation between theta and alpha (as can be seen from the power spectra in Figure 2, there is almost no periodic power in the alpha range in Front EW channels in B, and vice versa there's no periodic power in the theta range in Back BL channels in D).

Replicating what we found during resting EEG in the same participants (Snipes et al., 2023), extended wake increased the total number of theta bursts (Figure 2A) and decreased alpha bursts (Figure 2C) across all channels. At BL, theta bursts were on average 0.80 s [0.75, 0.83] long, with amplitudes of 15 µV [13, 16], and during EW they were 0.86 s [0.78, 0.88] long, and 18 µV [15, 19]. Alpha bursts were 0.57 s [0.50, 0.60] at BL and 0.56 s [0.49, 0.60] during EW, with amplitudes of 13 µV [11, 14] and 14 µV [12, 15] respectively.

On average, removing theta bursts reduced theta periodic power by 57% [40, 79] and removing alpha bursts reduced alpha periodic power by 100% [98, 102]. Increases > 100% were due to minor fitting imperfections of the aperiodic signal, visible only when virtually no periodic power remained. Therefore, the

---

[1] *This is a new criterion, each cycle is centred on the negative peak, then correlated with its two neighbouring cycles, and the smallest correlation was considered.*

burst detection successfully captured virtually all of the alpha oscillatory activity, and the majority of the theta oscillatory activity.

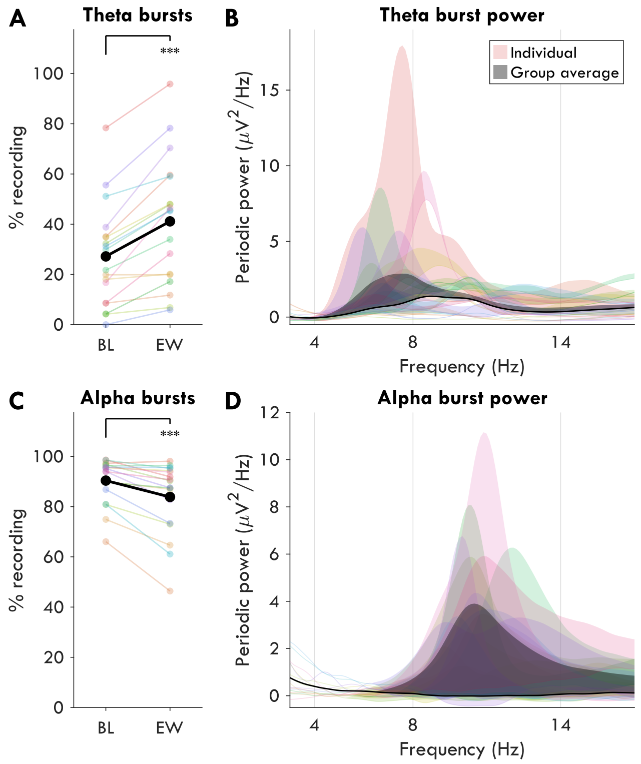

Figure 2: Burst detection. A: Percentage of the task recording characterized by theta at baseline (BL) and after extended wakefulness (EW). Thin colored lines indicate individual participants, black lines indicate the group average. Paired t-tests were conducted with α = 5%, such that: * p < .05, ** p<.01, *** p < .001. **B**: Periodic power spectrum from frontal channels during EW due to theta bursts. Periodic power refers to the power spectrum related to oscillatory activity, once the aperiodic 1/f signal has been subtracted. For each patch, the bottom line indicates the periodic power calculated from the EEG once theta bursts had been cut out, and the colored patch reflects periodic power of the EEG with theta bursts included (the bigger the patch, the more theta activity was in the recording). The colored patches are individual participants, and the black patch is the average across participants. If the bottom line of a patch is at 0, it means that all the oscillatory activity present in the EEG was captured by the burst detection. Channels included were the front region of interest described in Snipes et al. (2022). **C-D:** Same as A-B, but for alpha during BL, from posterior channels. Frontal channels during EW for theta and posterior channels during BL for alpha were chosen because these channels and recording times were when the most theta and alpha bursts were detected, respectively (Snipes et al., 2023).

Comparing wavelet time-frequency analysis

Given that the results in main Figure 3 contradict previous findings, we examined whether this was due to the difference in analysis method (cycle-by-cycle analysis vs time-frequency analysis) or paradigm (well-rested vs soporific conditions). We performed a traditional time-frequency analysis using wavelets (Figure 3).

Just after stimulus onset, for both fast and slow trials, there is an early increase in low frequencies and decrease in high frequencies. The low-frequency increase is, in all likelihood, merely the event related potential (ERP) triggered by the stimulus and subsequent response, which is why it does not appear in the burst analysis of main Figure 3. A ~1 s response reflects only a single cycle of a 1 Hz oscillation, and a ~0.25 s response reflects one cycle of a 5 Hz response; therefore the effect is not related to oscillations

per se. The beta-gamma (>15 Hz) decrease in power is likely the “event-related desynchronization” that happens first for fastest frequencies, then alpha, and finally in delta. This is then followed first by a rebound in beta/gamma, then in alpha.

Anticipating fast trials at BL, there is a decrease in all frequencies *except* alpha, with the largest effect in delta. This can be explained by simultaneous changes in periodic and aperiodic EEG activity (Donoghue et al., 2020). The larger decrease in the delta band compared to beta and gamma could be explained by aperiodic slopes being shallower before fast trials, counteracting the increased alpha periodic power due to the higher proportion of alpha bursts. Shallower aperiodic slopes reflecting higher vigilance is compatible with prior studies finding slope steepness to increase with increasing sleep depth, disorders of consciousness, and anaesthesia (Colombo et al., 2019, 2023; Maschke et al., 2023; Schneider et al., 2022).

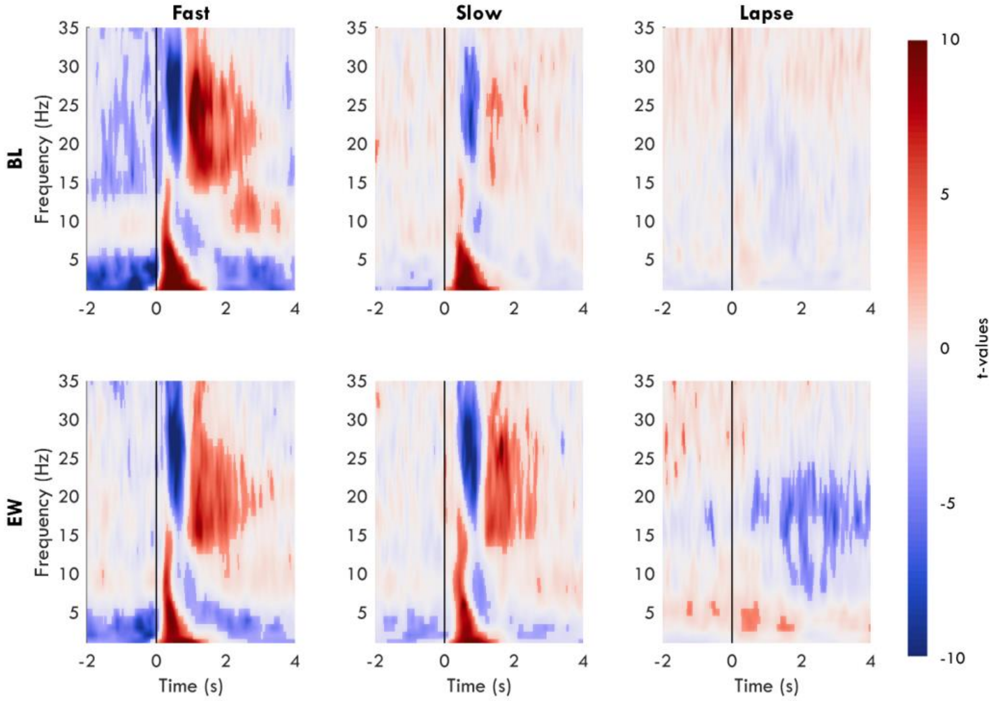

Figure 3: Time-frequency analysis locked to stimulus onset for BL fast trials (top) and EW lapse trials (bottom). Power values were log-transformed and compared to the session average, then averaged across all channels, with colour reflecting t-values such that red indicates higher than average power for that frequency at that timepoint. A pale mask covers values that were not statistically significant following FDR correction. Trials were *not* corrected for eye-closures.

REFERENCES

Cole, S., & Voytek, B. (2019). Cycle-by-cycle analysis of neural oscillations. *Journal of Neurophysiology, 122*(2). https://doi.org/10.1152/JN.00273.2019

Colombo, M. A., Comanducci, A., Casarotto, S., Derchi, C.-C., Annen, J., Viganò, A., Mazza, A., Trimarchi, P. D., Boly, M., Fecchio, M., Bodart, O., Navarro, J., Laureys, S., Gosseries, O., Massimini, M., Sarasso, S., & Rosanova, M. (2023). Beyond alpha power: EEG spatial and spectral gradients robustly stratify disorders of consciousness. *Cerebral Cortex, 33*(11), 7193–7210. https://doi.org/10.1093/cercor/bhad031

Colombo, M. A., Napolitani, M., Boly, M., Gosseries, O., Casarotto, S., Rosanova, M., Brichant, J.-F., Boveroux, P., Rex, S., Laureys, S., Massimini, M., Chieregato, A., & Sarasso, S. (2019). The spectral exponent of the resting EEG indexes the presence of consciousness during unresponsiveness induced by propofol, xenon, and ketamine. *NeuroImage, 189*, 631–644. https://doi.org/10.1016/j.neuroimage.2019.01.024

Donoghue, T., Haller, M., Peterson, E. J., Varma, P., Sebastian, P., Gao, R., Noto, T., Lara, A. H., Wallis, J. D., Knight, R. T., Shestyuk, A., & Voytek, B. (2020). Parameterizing neural power spectra into periodic and aperiodic components. *Nature Neuroscience, 23*(12), Article 12. https://doi.org/10.1038/s41593-020-00744-x

Maschke, C., Duclos, C., Owen, A. M., Jerbi, K., & Blain-Moraes, S. (2023). Aperiodic brain activity and response to anesthesia vary in disorders of consciousness. *NeuroImage, 275*, 120154. https://doi.org/10.1016/j.neuroimage.2023.120154

Schneider, B., Szalárdy, O., Ujma, P. P., Simor, P., Gombos, F., Kovács, I., Dresler, M., & Bódizs, R. (2022). Scale-free and oscillatory spectral measures of sleep stages in humans. *Frontiers in Neuroinformatics, 16*. https://www.frontiersin.org/articles/10.3389/fninf.2022.989262

Snipes, S., Krugliakova, E., Jaramillo, V., Volk, C., Furrer, M., Studler, M., LeBourgeois, M., Kurth, S., Jenni, O. G., & Huber, R. (2024). *Wake EEG oscillation dynamics reflect both sleep need and brain maturation across childhood and adolescence* (p. 2024.02.24.581878). bioRxiv. https://doi.org/10.1101/2024.02.24.581878

Snipes, S., Krugliakova, E., Meier, E., & Huber, R. (2022). The theta paradox: 4-8 Hz EEG oscillations reflect both sleep pressure and cognitive control. *Journal of Neuroscience*. https://doi.org/10.1523/JNEUROSCI.1063-22.2022

Snipes, S., Meier, E., Meissner, S. N., Landolt, H.-P., & Huber, R. (2023). How and when EEG reflects changes in neuronal connectivity due to time awake. *iScience, 26*(7). https://doi.org/10.1016/j.isci.2023.107138
