# Supplementary material for: Extended wakefulness alters the relationship between EEG oscillations and performance in a sustained attention task
Source: J Sleep Res. 2024 May 5;33(6):e14230. doi: 10.1111/jsr.14230 (PMC11596987; doi:10.1111/jsr.14230)
Supplement: Supplementary file 2 — DATA S2 Supporting Information. [file JSR-33-e14230-s002.pdf]

Screening Questionnaire

OVERVIEW

The following questions were presented in the online entrance questionnaire. The entrance questionnaire is composed of separate sections. Throughout the questionnaire, if responders were eligible for the experiment, they were informed as soon as they finished the section that disqualified them. At no point were responders specifically informed about what answer they gave that no longer rendered them eligible. This is to avoid responders "trying again" and changing their answers, but also to avoid any appearance of judgement or diagnosis.
After each section, if any answer matched our exclusion criteria, responders were redirected to the Not Eligible page. If they were eligible according to all sections, they were asked to confirm that all their answers were accurate, then redirected to the Eligible page. In particular, they were provided with a contact email and a 3-word code. This code is the only link between a given participant and their answers, thus making the entire questionnaire fully anonymous.
All questionnaires will be provided in English and German to ensure the participant answers accurately.

Symbols throughout the document:
The following characters indicate what kind of answers participants could provide:
" • " this indicates multiple choices where participants can only choose 1 answer.
" [] " indicate open numeric answers.
" {} " indicate open text answers.

The following symbols indicate which answers will exclude the applicant:
*" * " indicates the answers that would by itself exclude the responder.*
" ~ " indicates answers that there needs to be a minimum number of such answers before the responder is excluded.

All text in italics is metainformation about the screening questionnaire and qualifying/disqualifying answers.

SCREENING QUESTIONS

1. Sensitive Screening Checklist

Please answer honestly the following yes or no questions about your health and drug use. This is important to exclude responders who may react badly to the conditions of the experiment, or are otherwise considered “at risk”. Your answers will not be saved. In this section we ask if you have any medical conditions or take any medications; if you are unsure about the meaning of these terms, just click on them, and you will be redirected to the relevant Wikipedia page.

1.1. Do you have sensitive skin? / Haben Sie empfindliche Haut?
This means that cleaning without gloves results in skin irritations, you need a special sun block, you have skin eczemas, allergies, psoriasis (long lasting red, dry, itchy skin), or problems with acne, etc.
Dies bedeutet, dass Reinigung ohne Handschuhe zu Hautreizungen führt, Sie einen speziellen Sonnenschutz benötigen, Hautekzeme, Allergien Psoriasis (lang anhaltende rote, trockene, juckende Haut) oder Probleme mit Akne usw. haben.

- Yes *
- No

1.2. Have you had major surgery in the past 3 months? / Hatten Sie in den letzten 3 Monaten eine grössere Operation?
Requiring full-body anaesthesia.
Unter Vollnarkose.

- Yes *
- No

1.3. Have you ever had brain surgery? / Hatten Sie jemals eine Operation am Gehirn?

- Yes *
- No

1.4. Have you ever been diagnosed with (or strongly suspected of having) a neurological/psychiatric disorder, or suffered brain damage? / Wurde bei Ihnen jemals eine neurologische / psychiatrische Störung diagnostiziert (oder wurde dies stark vermutet) oder wurde Ihr Gehirn geschädigt?
Examples include, but are not limited to: anorexia, ADHD, anxiety, autism, bipolar disorder, brain haemorrhage/aneurysm, brain malformations, bulimia, cerebral palsy, major concussion, cluster headaches, frequent and severe migraines, depression, dystonia, epilepsy, intracranial pressure, multiple sclerosis, phobias, psychosis, PTSD, schizophrenia, stroke, tumours, etc.
Z.B.: Anorexie, ADHS, Angstzustände, Autismus, bipolare Störung, Gehirnblutung / -aneurysma, Gehirnfehlbildungen, Bulimie, Zerebralparese, schwere Gehirnerschütterung, Clusterkopfschmerzen, häufige und schwere Migräne, Depression, Dystonie, Epilepsie, erhöhter Hirndruck, Multiple Sklerose, Phobien, Psychosen, PTBS, Schizophrenie, Schlaganfall, Tumoren usw.

- Yes *
- No

1.5. Have you ever been diagnosed or strongly suspected of having a learning disability? / Wurde bei Ihnen jemals eine Lernschwäche diagnostiziert oder stak vermutet?
Examples include: dyslexia, discalculia, dysgrafia, etc.
Z.B.: Legasthenie, Diskalkulie, Dysgrafie usw.

- Yes *
- No

1.6. Do you have any hearing impairments in either ear? / Haben Sie Hörstörungen in einem Ohr?

Examples include: minor deafness, tinnitus, cochlear implants (anything requiring hearing assistance device), etc.
Z.B.: leichte Taubheit, Tinnitus, Cochlea-Implantate (alles, was ein Hörgerät erfordert) usw.

- Yes *
- No

1.7. Can you see clearly from both eyes? / Können Sie mit beiden Augen gut sehen?
You can answer "yes" if you can see clearly while wearing contact lenses or glasses. Please answer "no" for color blindness.
Antworten Sie „ja", wenn Sie mit Kontaktlinsen oder Brillen klar sehen können. Bitte antworten Sie mit "Nein" für Farbenblindheit.

- Yes
- No *

1.8. Have you suffered from a serious infectious disease in the past 3 years, or are still affected by one? / Haben Sie in den letzten 3 Jahren an einer schweren Infektionskrankheit gelitten oder sind immer noch davon betroffen?
These are diseases that required you to take medication for a prolonged period (>2 weeks) and/or that prevent you from working/studying 100% of the time.
Examples: Lyme disease, AIDS/HIV, syphilis, FSME (tick encephalitis), parasites, dengue, malaria, TB, Hepatitis C, measles, diphtheria, legionnaires.
Gemeint sind Krankheiten, bei denen Sie über einen längeren Zeitraum (> 2 Wochen) Medikamente einnehmen müssen und / oder die Sie daran hindern, 100% zu arbeiten / zu studieren.
Z.B.: Lyme-Borreliose, AIDS / HIV, Syphilis, FSME (Zeckenenzephalitis), Parasiten, Dengue-Fieber, Malaria, TB, Hepatitis C, Masern, Diphtherie, Legionellen.

- Yes *
- No

1.9. Do you suffer regularly from fatigue, either physical or mental? / Leiden Sie regelmäßig unter physischer oder psychischer Müdigkeit?
This means you tire easily throughout the day, so much so that it seriously impacts your work and social life.
Das bedeutet, dass Sie am Tag schnell müde werden, was Ihre Arbeit und Ihr soziales Leben ernsthaft beeinträchtigt.

- Yes *
- No

1.10. Have you fainted or lost consciousness at least once in the past year for more than 5 minutes? / Haben Sie im letzten Jahr mindestens einmal mehr als 5 Minuten lang das Bewusstsein verloren?

- Yes *
- No

1.11. Do you regularly faint or suffer from dizziness? / Fühlen Sie sich regelmäßig schwindelig oder fallen in Ohnmacht?

- Yes *
- No

1.12. Do you have any impairment to your hands/fingers on either hand that make typing challenging? / Haben Sie eine Beeinträchtigung Ihrer Hände / Finger, die das Tippen schwierig macht?
This can be caused by anything, both temporary or permanent.
Dies kann vorübergehend oder dauerhaft sein.

- Yes *
- No

1.13. Do you have any other physical impairment, medical condition or disability? / Haben Sie eine andere körperliche Beeinträchtigung, Krankheit oder Behinderung?

Examples include but are not limited to: autoimmune disorders, metabolic disorders, paraplegia (impairment of legs), hormonal disorders, tumours (also benign), cardiovascular problems (e.g. past heart attack, angina, weak heart, congenital heart defects), diabetes, obesity, kidney problems, severe scoliosis (bent spine), organ transplant, severe asthma, lung deficiencies, third degree burns, chronic pain, anaemia (iron deficiency), thyroid problems, strabismus.

Z.B.: Autoimmunerkrankungen, Stoffwechselerkrankungen, Querschnittslähmung (Beeinträchtigung der Beine), hormonelle Störungen, Tumoren (auch gutartig), Herz-Kreislauf-Probleme (z. B. Herzinfarkt in der Vergangenheit, Angina, schwaches Herz, angeborene Herzfehler), Diabetes, Fettleibigkeit, Nierenprobleme, schwere Skoliose (gebogener Rücken), Organtransplantation, schweres Asthma, schwere Lungenerkrankungen, Verbrennungen dritten Grades, chronische Schmerzen, Anämie (Eisenmangel), Schilddrüsenprobleme, Schielen.

- Yes *
- No

1.14. Are you currently or have recently been required to wear a cast or long-term bandage? / Müssen Sie derzeit einen Gipsverband tragen oder hatten kürzlich einen Langzeitverband?

For example from a broken arm or pulled tendon. If the same amount of time you were wearing the cast/bandage has already passed without it, and you can freely move as before, you can answer “no”.

Zum Beispiel von einem gebrochenen Arm oder einer überdehnten Sehne. Wenn die gleiche Zeit, die Sie mit dem Verband verbracht haben, bereits ohne vergangen ist und Sie sich wie zuvor frei bewegen können, können Sie mit „Nein“ antworten.

- Yes *
- No

1.15. Do you currently take any prescription medication? / Nehmen Sie derzeit verschreibungspflichtige Medikamente ein?

This is any pill, tablet, syrup, or other that required a doctor’s prescription to obtain from the pharmacy.
The only exceptions are: contraception, creams for limited external use.

Dies ist jede Pille, Tablette, Sirup oder anderer Stoff, für den ein ärztliches Rezept in der Apotheke erforderlich ist.
Die einzigen Ausnahmen sind: Empfängnisverhütung, Cremes zur eingeschränkten äußerlichen Anwendung.

- Yes *
- No

1.16. Have you taken prescription medication in the past month? / Haben Sie im letzten Monat verschreibungspflichtige Medikamente eingenommen?

- Yes *
- No

1.17. Have you ever used recreational drugs regularly, i.e. more than once per week? / Haben Sie jemals regelmäßig Drogen konsumiert, d. h. mehr als einmal pro Woche?

Examples include but are not limited to: marijuana, heroin, opium, cocaine, meth, amphetamines, MDMA, GHB, G, speed, ecstasy, LSD, mescaline, psilocybin (i.e. “magic mushrooms”), steroids.

Z.B.: Marihuana, Heroin, Opium, Kokain, Meth, Amphetamine, MDMA, GHB, G, speed, Ecstasy, LSD, Mescalin, Psilocybin (d. h. "magic mushrooms"), Steroide.

- Yes *
- No

1.18. Have you ever suffered an addiction requiring rehabilitation to drugs, gambling, or other? / Hatten Sie jemals eine Suchterkrankung (z.B. Drogen, Glücksspiel, ect), die eine Rehabilitation erforderte?

- Yes *
- No

1.19. Have you ever experienced withdrawal from a drug? / Hatten Sie jemals Entzugssymptome von Drogen?

Common examples include nicotine/cigarettes and opioids/painkillers.

Häufige Beispiele sind Nikotin / Zigaretten und Opioide / Schmerzmittel.

- Yes *
- No

1.20. Have you ever suffered from alcohol abuse? / Haben Sie jemals unter Alkoholmissbrauch gelitten?

This means you were drinking regularly until severely drunk several times per week or more.

Dies bedeutet, dass Sie mehrmals pro Woche stark betrunken waren.

- Yes *
- No

1.21. Have you ever suffered from a sleep disorder? / Haben Sie jemals an einer Schlafstörung gelitten?

Examples include: sleep apnoea, severe snoring, narcolepsy, parasomnia, sleep paralysis, cataplexy (sudden muscle weakness), fainting bouts, chronic fatigue, hypersomnia (excessive time spent sleeping), insomnia, restless leg syndrome.

Z.B.: Schlafapnoe, starkes Schnarchen, Narkolepsie, Parasomnie, Schlaflähmung, Kataplexie (plötzliche Muskelschwäche), Ohnmacht, chronische Müdigkeit, Hypersomnie (übermäßige Schlafenszeit), Schlaflosigkeit, Restless Leg Syndrom.

- Yes *
- No

1.22. Do you regularly (at least once per week) take medication to help you sleep or that impact your sleep? / Nehmen Sie regelmäßig (mindestens einmal pro Woche) Medikamente ein, die Ihnen beim Schlafen helfen oder sich auf Ihren Schlaf auswirken?

Examples: Zolpidem (Ambien®, Stilnox®), benzodiazepines (Temesta®, Seresta®, Xanax®, Dormicum®), antidepressives (Remeron®, Trittico®), antihistamines (allergy drugs such as Sanalepsi N®, Benocten®)

Z.B.: Zolpidem (Ambien®, Stilnox®), Benzodiazepine (Temesta®, Seresta®, Xanax®, Dormicum®), Antidepressiva (Remeron®, Trittico®), Antihistaminika (Medikamente gegen Allergien, wie Sanalepsi N®, Benocten®)

- Yes *
- No

1.23. Do you take any medication as a stimulant? / Nehmen Sie Medikamente als Stimulantien ein?

Examples: Adderall®, methylphenidate (Ritalin®), modafinil (Provigil®)

Z.B.: Adderall®, Methylphenidat (Ritalin®), modafinil (Provigil®)

- Yes *
- No

1.24. Have you ever suffered from difficult sleep for periods longer than a month in the past year? / Haben Sie im vergangenen Jahr länger als einen Monat unter Schlafstörungen gelitten?

This means you had trouble falling asleep, trouble staying asleep, waking up frequently, woke up not feeling rested, etc.

Gemeint sind Probleme beim Einschlafen, Durchschlafen, häufiges Aufwachen, Aufwachen ohne genügende Erholung usw.

- Yes *
- No

1.25. Do you currently suffer from daytime sleepiness? **/ Leiden Sie derzeit unter Tagesmüdigkeit?**
This means you regularly feel sleepy most of the day, for most days.
Dies bedeutet, dass Sie sich an den meisten Tagen den ganzen Tag über regelmäßig schläfrig fühlen.

- Yes *
- No

1.26. Do you suffer from seasonal changes in mood or sleepiness, enough to disrupt your ability to work or study normally? **/ Leiden Sie unter saisonalen Stimmungsschwankungen oder erhöhter Schläfrigkeit, die Ihre Fähigkeit normal zu arbeiten oder zu lernen beeinträchtigen?**

- Yes *
- No

1.27. Are you pregnant? **/ Sind Sie schwanger?**

- Yes *
- No

1.28. Do you have children younger than 5? **/ Haben Sie Kinder unter 5 Jahren?**

- Yes *
- No

1.29. Have any of the following affected you in the past month, enough to impact your sleep, restfulness, or mood/psychological wellbeing? **/ Haben Sie im letzten Monat eine der folgenden Situationen erlebt, die Ihren Schlaf, Ihre Ruhe oder Ihre Stimmung bzw. Ihr psychisches Wohlbefinden beeinträchtigten?**

Death of a close friend or relative / Tod eines engen Freundes oder Verwandten	Yes*	No
Severe medical illness, treated or untreated / Schwere medizinische Erkrankung, behandelt oder unbehandelt	Yes*	No
A serious accident (e.g. a car crash) / Ein schwerer Unfall (z. B. ein Autounfall)	Yes*	No
Taking care of a seriously ill friend or relative / Betreuung eines schwerkranken Freun-des oder Verwandten	Yes*	No
Yes, but I'd rather not say / Ja, aber ich möchte es nicht sagen	Yes*	No
Other / Andere	Yes*	No

2. Standardized Screening Questionnaires

2.1. Pittsburgh Sleep Quality Index (PSQI)

This is a validated test designed to identify poor sleep quality in both healthy and patient populations. The test produces a score from 0 to 21; responders with a PSQI score higher than 6 were excluded. Official translations exist and are therefore not reported here.

2.1.1. During the past month, what time have you usually gone to bed at night?
Clock time: []

2.1.2. During the past month, how long (in minutes) has it usually taken you to fall asleep each night?
Minutes: []

2.1.3. During the past month, what time have you usually gotten up in the morning?
Clock time: []

2.1.4. During the past month, how many hours of actual sleep did you get at night?
This may be different than the number of hours you spent in bed.
Hours: []

2.1.5. During the past month, how often have you had trouble sleeping because you...

Cannot go to sleep within 30 minutes	Not during the past month	Less than once a week	Once or twice a week	Three or more times per week
Wake up in the middle of the night or early morning				
Have to get up to use the bathroom				
Cannot breath comfortably				
Cough or snore loudly				
Feel too cold				
Feel too hot				
Have bad dreams				
Have pain				
other				

2.1.6. During the past month, how would you rate your sleep quality overall?
- Very good
- Fairly good
- Fairly bad
- Very bad

2.1.7. During the past month, how often have you taken medicine to help you sleep ("over the counter" or prescribed)?
- Not during the past month
- Less than once a week
- Once or twice a week
- Three or more times per week

2.1.8. During the past month, how often have you had trouble staying awake while driving, eating meals, or engaging in social activity?
- Not during the past month
- Less than once a week
- Once or twice a week

- Three or more times per week

2.1.9. During the past month, how much of a problem has it been for you to keep enough enthusiasm to get things done?

- No problem at all
- Only a very slight problem
- Somewhat of a problem
- A very big problem

2.1.10. Do you have a bed partner or room mate?

- No bed partner or room mate
- Partner/room mate in other room
- Partner/room mate in same room, but not same bed
- Partner in same bed

2.2. Munich Chronotype Questionnaire (reduced)

This is a questionnaire with a specific algorithm for determining chronotype. From the answers, the chronotype score will be on a scale from 1 to 12. Based on a prior established distribution, participants with MCTQ scores outside 2-6.5 were excluded. Additionally, participants with a discrepancy larger than 3 hours from weekdays to weekends were excluded.

2.2.1. How many working/school days do you have per week? []

On school/working days:

> **2.2.2. At what time do you turn the light off to go to sleep (including putting phone or tablet down)?** []
> **2.2.3. How many minutes do you need to fall asleep?** []
> **2.2.4. At what time do you wake up?** []

On free days:

> **2.2.5. At what time do you turn the light off to go to sleep?** []
> **2.2.6. How many minutes do you need to fall asleep?** []
> **2.2.7. At what time do you wake up?** []

3. Study-Specific Questionnaires

3.1. Wake Behaviour

3.1.1. Age / Alter: []
Participants under 18 or over 25 were excluded.

3.1.2. Height / Grösse: []
3.1.3. Weight / Gewicht: []
These are to calculate the BMI of the participants, and ensure they are in a healthy range (18.5-30). Participants that were over or underweight were excluded.

3.1.4. Do you smoke, vape, or use nicotine patches? / Rauchen, vapen oder benutzen Sie Nikotinpflaster?

- Never / Noch nie
- Once per month or less / Einmal pro Monat oder weniger
- Once per week or less / Einmal pro Woche oder weniger *
- Once per day / Einmal am Tag *
- Several times per day / mehrmals täglich *

3.1.5. Have you ever smoked regularly (>1 cigarette per day) previously? / Haben Sie schon einmal regelmäßig geraucht (> 1 Zigarette pro Tag)?

- Yes *
- No

3.1.6. In the following table, indicate for each row how often you drink a given amount of alcohol in a single occasion. / Geben Sie in der folgenden Tabelle für jede Zeile an, wie oft Sie bei einer bestimmten Gelegenheit eine bestimmte Menge Alkohol trinken.

	Never	Once per month or less	Once per week or less	A couple times per week / Ein paar mal pro Woche	Every day / Jeden Tag
One drink / Ein Getränk					*
2-3 drinks / 2-3 Getränke					*
4-6 drinks				*	*
>6 drinks			*	*	*

3.1.7. Do you regularly drink coffee or other caffeinated beverages, especially during the week? / Trinken Sie regelmäßig Kaffee oder andere koffeinhaltige Getränke, besonders während der Woche?
Indicate how many servings per day for each category.
Geben Sie an, wie viele Portionen pro Tag für jede Kategorie.

	Less than 1 per day / Weniger als 1 pro Tag	1-2 servings per day / Portionen pro Tag	3 servings per day	4 or more servings per day
Coffee / Kaffee			*	*
Energy drinks (e.g. Red Bull)			*	*

Black/green tea / schwarzer / grüner tee				*
Caffeinated soft drinks (e.g. Coca Cola) / Koffeinhaltige Erfrischungsgetränke				*

This is to exclude responders who are heavy consumers of caffeine. Furthermore, if the sum of drinks is 4 or more, the responder was excluded.

3.1.8. Do you "need" coffee in the morning before you feel awake? / Brauchen Sie morgens Kaffee, um sich wach zu fühlen?

- Yes *
- No
- Sometimes / Manchmal

3.1.9. Do you ever regularly take some other food, drink, or medication to stay awake? / Nehmen Sie regelmäßig etwas zu essen, zu trinken oder Medikamente ein, um wach zu bleiben?

- Yes: {} *
- No

3.1.10. Over the past month, how stressed did you feel on a regular basis? / Wie gestresst haben Sie sich im letzten Monat regelmäßig gefühlt?

- Vacation-level relaxed / Urlaubsniveau entspannt
- Relaxed / entspannt
- Some stress / Etwas Stress
- A lot of stress / Sehr viel Stress *

3.1.11. In general, how vulnerable are you to stress? / Wie anfällig sind Sie im Allgemeinen für Stress?

- I'm almost never stressed / Ich bin fast nie gestresst
- Only important things stress me out / Nur wichtige Dinge belasten mich
- Some things stress me out / Einige Dinge belasten mich
- A lot of things stress me out / Viele Dinge belasten mich *

3.1.12. How often do you travel across 2 or more time zones? / Wie oft reisen Sie durch zwei oder mehr Zeitzonen?

- Never
- Rarely, a couple times per year / Selten, ein paar Mal im Jahr
- Occasionally, 3-4 times per year / Gelegentlich, 3-4 mal pro Jahr
- Around once every 2 months / Ungefähr alle 2 Monate *
- Almost every month / Fast jeden Monat *
- More than once per month / Mehr als einmal pro Monat *

3.2. Sleep Behaviour

3.2.1. On week days, at what time would you prefer to wake up? / Wann möchten Sie an Wochentagen aufwachen? []
If the answer here is >2h then actual wakeup time, participant were excluded.

3.2.2. On week days, when would you prefer to go to sleep? / Wann möchten Sie an Wochentagen schlafen gehen? []

3.2.3. How often do you have to set an alarm 2 hours before your preferred wakeup time? / Wie oft müssen Sie 2 Stunden vor Ihrer bevorzugten Weckzeit einen Wecker stellen?
- Never
- Sometimes, once per month or less / Manchmal, einmal im Monat oder weniger
- 2-4 times per month
- More than once per week *

3.2.4. Have you had a schedule in the past 5 years that required you to sleep more than 3 hours off from your preferred sleep window? / Hatten Sie in den letzten 5 Jahren einen Zeitplan, nach dem Sie mehr als 3 Stunden von Ihrem bevorzugten Schlaffenster entfernt schlafen mussten?
This includes night shifts, jobs you had to wake up extremely early to get to, or go to sleep much later than preferred. A "job" means this lasted 1 month or more.
Dazu gehören Nachtschichten, Jobs, bei denen man extrem früh aufstehen musste, oder Jobs, bei denen man viel später als gewünscht schlafen ging. Ein "Job" bedeutet, dass dieser 1 Monat oder länger dauerte.
- Never
- Once, more than 1 year ago / Einmal vor mehr als einem Jahr
- Once, this year / Einmal in diesem Jahr *
- More than once / Mehr als einmal *

3.2.5. Do you go to bed at a regular time and get up at a regular time even if you don't have to? / Gehen Sie zu regelmässigen Zeiten ins Bett und stehen Sie zu regelmässigen Zeiten auf, auch wenn Sie das nicht müssen?
- Never *
- Not really / Nicht wirklich
- Sometimes
- Often
- Always

3.2.6. Do you nap? / Machen Sie Nickerchen?
- Less than once per month
- Once per week or less
- More than once per week *
- Every day *

3.2.7. Are you the sort of person who can easily miss out on a night of sleep? / Sind Sie die Art von Person, die leicht auf eine Nacht Schlaf verzichten kann?
- Definitely not / Definitiv nicht
- Not really
- Sometimes
- Fairly easy / Ziemlich einfach *
- Definitely / Definitiv *

3.2.8. How do you react to working late in the night? / Wie reagieren Sie darauf, spät in der Nacht zu arbeiten?
- I can't do it / Ich kann es nicht
- I dislike doing it / Ich mag es nicht
- I sometimes do it / Ich mache es manchmal
- I have no problem doing it / Ich habe kein Problem damit
- It's my most productive time to work / Es ist meine produktivste Zeit zu arbeiten *

3.2.9. How often have you spent 20 or more hours awake? / Wie oft haben Sie 20 oder mehr Stunden wach verbracht?
This would mean that you stayed awake until the early morning hours.
Dies würde bedeuten, dass Sie bis in die frühen Morgenstunden wach blieben.
- Never
- Less than twice per year
- 2-5 times per year
- around once every month or two *
- more than once per month *

3.2.10. How many hours of sleep do you prefer to have? / Wie viele Stunden schlafen Sie am liebsten?
[]
If less than 6 or more than 10, excluded.

3.2.11. How often do you sleep less than 5 hours? / Wie oft schlafen Sie weniger als 5 Stunden?
- Never
- Less than twice per year
- 2-6 times per year
- around once per month
- more than once per month *

3.2.12. When you do, does it affect your: / Wenn Sie dies tun, wirkt sich dies auf Folgendes aus:
- ✓ mood / Stimmung
- ✓ physical performance / körperliche Leistungsfähigkeit
- ✓ mental performance / mentale Leistungsfähigkeit
- ✓ safety / Sicherheit
- ✓ clumsiness / Ungeschicklichkeit
- ✓ sleepiness / Schläfrigkeit
- ✓ energy levels / Energieniveaus
- ✓ All of the above / Alles oben Genannte

if none of the above were clicked, person was excluded (meaning they were not vulnerable to sleep loss).

3.2.13. Are you particular about your pillow? / Brauchen Sie ein bestimmtes Kissen?
- I can only sleep on my own pillow / Ich kann nur auf meinem eigenen Kissen schlafen *
- I sleep poorly on most pillows / Ich schlafe auf den meisten Kissen schlecht
- I prefer some pillows over others / Ich bevorzuge einige Kissen gegenüber anderen
- I can sleep on most pillows / Ich kann auf den meisten Kissen schlafen
- I can sleep with no pillows / Ich kann ohne Kissen schlafen

This is an exclusion criteria for practical reasons; if someone is so delicate in their sleep, then in the lab they are not likely to fall asleep.

3.2.14. How much do the following disturb your sleep? / Wie sehr stören die folgenden Dinge Ihren Schlaf?

Different bed / Anderes Bett: not at all / just a little /somewhat / often~ / always*

Traffic (or other outside) noise / Verkehrslärm (oder sonstiger Außenlärm): not at all / just a little /somewhat / often / always~

Voices in other rooms / Stimmen in Nebenräumen: not at all / just a little /somewhat / often / always~
Noise from electronic devices / Geräusche von elektronischen Geräten: not at all / just a little /somewhat / often~ / always*
Bedroom temperature / Schlafzimmertemperatur: not at all / just a little /somewhat / often~ / always*
Seasons / Jahreszeiten: not at all / just a little /somewhat / often~ / always*
Light from window / Licht vom Fenster: not at all / just a little /somewhat / often / always~
LED lights in the room / LED Licht im Raum: not at all / just a little /somewhat / often~ / always~

If 3 or more of the ~ answers were chosen, then the participant was excluded.

3.2.15. Is there anything you "need" to do to make sure you have a good night sleep? / Gibt es irgendetwas, das Sie tun müssen, um einen guten Schlaf zu haben?

- Yes: {} *
- No

3.2.16. Do you ever talk in your sleep? / Sprechen Sie jemals im Schlaf?

- Never
- Very rarely, very little / Sehr selten, sehr wenig
- Once a week or so, a little / Einmal in der Woche oder so etwas
- Once a week or so, a lot / Einmal in der Woche oder so, viel *
- Almost every night / Fast jede Nacht *

3.2.17. Did you ever sleepwalk as an adult? / Haben Sie schon einmal als Erwachsener geschlafwandelt?

- Never
- Rarely *
- Sometimes *
- Regularly / Regelmässig *
- Almost every night *

3.2.18. Have you suffered from bad dreams in the past few months? / Haben Sie in den letzten Monaten unter schlechten Träumen gelitten?

- Never
- Rarely
- Once per month or less
- A couple times per week *
- Every night *

3.2.19. Do you have phases, several nights in a row, in which you have difficulty falling asleep? / Haben Sie Phasen von mehreren Nächten hintereinander, in denen Sie Schwierigkeiten haben einzuschlafen?

- Never/Rarely
- Less than once every 3 months
- Less than once per month
- More than once per month *
- Regularly *

3.2.20. How often do you wake up at night? / Wie oft wachen Sie nachts auf?

- Never
- Rarely
- Some nights, but not regularly / Manche Nächte, aber nicht regelmäßig
- Almost every night, just once / Fast jede Nacht, nur einmal *
- A couple times per night / Ein paar Mal pro Nacht *

- Many times per night *

3.2.21. Do you wake up to go to the bathroom at night? / Wachen Sie nachts auf, um auf die Toilette zu gehen?

- Never
- Rarely
- Some nights, but not regularly
- Almost every night, just once *
- A couple times per night or more / Ein paar Mal pro Nacht oder mehr *

3.2.22. Do you wake up to drink water at night?

- Never
- Rarely
- Some nights, but not regularly
- Almost every night, just once
- A couple times per night or more *

3.2.23. Do you consider yourself:

- A poor sleeper / Ein schlechter Schläfer *
- A light sleeper / Ein leichter Schläfer *
- An average sleeper / Ein durchschnittlicher Schläfer
- A deep sleeper / Ein tiefer Schläfer

3.2.24. Do you ever take supplements for improving sleep, such as melatonin or valerian? / Nehmen Sie jemals Nahrungsergänzungsmittel zur Verbesserung des Schlafes ein, wie Melatonin oder Baldrian?

- Never
- Less than once per month
- A couple times per month
- A couple times per week *
- Every night *

3.2.25. Do you take herbal medicine or homeopathy to help you sleep? / Nehmen Sie Kräutermedizin oder homöopathische Mittel ein, um beim Schlafen zu helfen?

- Never
- Less than once a month
- Less than once per week
- A couple times per week
- Every night

RESULTS PAGES

Confirm results page

The last step involved asking participants to confirm whether their answers were accurate and honest, and that they understood all the questions. This is to avoid saving data if someone is just playing around with the questionnaire, or if the participant reflects on the fact that they may have answered a little too distractedly.

We are a scientific research lab operating on a budget and dedicated to producing the highest quality research. Intentionally incorrect answers will lead to unintentionally false results. Science depends on you being truthful and thorough.

A) Please discard my questionnaire (for whatever reason) *
B) I'm not really sure about how accurate my answers were
C) I may have made mistakes, but they were unintentional and would be happy to correct them
D) I confirm that to the best of my knowledge, all the answers provided are correct

After answers B-D, participants were directed to the following pages.

INSTRUCTIONS FOR PARTICIPATION

For this experiment, we want to ensure a relatively uneventful sleep-wake rhythm 1 month prior to the experiment day. Please make sure that none of the following occur in the month before the main experiment, and if they do, please reschedule the appointment.

- Take any drugs or medication, including marijuana, allergy drugs, etc.
- Smoke, vape or use nicotine patches
- Get sick (e.g. flu) or seriously injured (e.g. broken arm)
- Travel across 2 or more time zones
- 5 or more consecutive days of poor sleep for whatever reason
- Sleep deprivation (staying awake longer than 20 hours)
- Sleep restriction (sleeping less than 6 hours a night for at least 3 consecutive nights)

If any of the following happen to you, please schedule the first appointment only after you have completely recovered:

- A cold or similar small illness (can still go to work)
- Vaccine (4-5 days after injection)
- Menstruation
- Exam
- Music festival or other exhausting event
- Long trip
- Terrible sleep for one night
